# Transient Electroosmotic Flow of Maxwell Fluids Through Soft Channels with High Surface Potentials

**DOI:** 10.3390/polym18131596

**Published:** 2026-06-26

**Authors:** Clara G. Hernández, Juan P. Escandón, Edson M. Jimenez, Juan R. Gómez, René O. Vargas, David A. Torres, Nicolas Ratkovich

**Affiliations:** 1 Departamento de Termofluidos, SEPI-ESIME Unidad Azcapotzalco, Instituto Politécnico Nacional, Ciudad de México 02250, Mexico; claragroblero@gmail.com (C.G.H.); ejimenez10.emjd@gmail.com (E.M.J.); jrkano@outlook.com (J.R.G.); rvargasa@ipn.mx (R.O.V.); 2 Programa Educativo de Química, Universidad Tecnológica de Tula-Tepeji, Tula de Allende 42830, Mexico; davidalejandro.torres@uttt.edu.mx; 3 Department of Chemical and Food Engineering, Universidad de los Andes, Bogotá 111711, Colombia; n.rios262@uniandes.edu.co

**Keywords:** transient electroosmotic flow, soft channel, polyelectrolyte layer, Maxwell fluid, high surface potentials, steric effect

## Abstract

This study analyzes the combined effects of non-Newtonian rheology and electrostatics on the transient electroosmotic flow of Maxwell fluids in soft channels. The walls of the rigid channels are hydrophobic, ionically charged, and coated with a polyelectrolyte layer (PEL). This design is intended to regulate both the surface electric potential and the flow velocity. The mathematical model is based on modified Poisson–Boltzmann and momentum equations, which are solved numerically using a one-dimensional (1D) approach. The results indicate that high potentials, exceeding the Debye–Hückel limit, are achieved under conditions of thick polyelectrolyte layers, high surface charge density, and a higher concentration of fixed charges compared to the electrolyte ionic concentration. In this regime, steric effects increase the electric potential; however, this potential increase is limited by the formation of a Donnan potential. The hydrodynamic analysis demonstrates that the velocity magnitude is influenced not only by the wall potential but also by the spatial distribution of free charge density and electroosmotic force, which, in turn, are affected by steric effects. Additionally, changing the polarity and concentration of fixed charge in the PEL produces asymmetric flows, and while hydrodynamic slip enhances velocity, the drag parameter reduces it. Finally, the dimensionless parameters that control the time required to dampen the oscillatory flow induced by viscoelastic effects and reach steady-state are mainly the relaxation time, the drag parameter, the PEL thickness, and the electrokinetic parameter of the PEL, while the surface charge density and the external pressure gradient exert a comparatively minor influence.

## 1. Introduction

Microfluidics and nanofluidics study fluid flow in channels ranging from several microns to several hundreds of microns (microchannels) [1,2,3] and from a few nanometers to 1000 nm (nanochannels) [4,5,6,7,8,9], handling small fluid volumes ranging from $10^{-21}$ to $10^{-6}$ L [1,8,10,11]. These disciplines are widely used to analyze fluid samples in chemical [8,12], biological [9,13], and medical [14,15] research. Integration of techniques from these fields is implemented in devices such as lab-on-a-chip systems [16,17], microelectromechanical systems (MEMSs) [18], nanoelectromechanical systems (NEMSs) [9,19], micro total analysis systems (μTASs) [20], and point-of-care (POC) devices [21,22].

Electroosmotic flow is commonly used for fluid manipulation in micro- and nanofluidic systems [23,24]. This pumping mechanism is based on the formation of an electric double layer (EDL) near a charged solid–liquid interface, where oppositely charged ions from an electrolyte solution accumulate and migrate under an external electric field, thereby inducing fluid motion [25,26]. Typically, when bare channel walls are in contact with an electrolyte, they acquire a surface electric charge and form an EDL. In these cases, the electric potential, also known as the zeta potential, at the shear plane between the charged surface and the electrolyte solution controls the flow [25,27,28]. However, surface electric potential and electroosmotic flow can also be controlled by coating channel walls with oils [29], surfactants [30,31], or polymers [32,33,34,35].

Polyelectrolytes are polymers that carry ionizable groups with positive or negative charges [36]. When a surface is coated with a polyelectrolyte layer (PEL), polymer brushes (polymer chains) with fixed ionic charges are grafted onto it [37,38]. In electroosmotic flows, polyelectrolyte layers (PELs) on the channel walls (often referred to as soft channels) modify fluid motion by introducing hydrodynamic resistance through polyelectrolyte brushes and by attracting counterions, thereby altering the electrostatic potential [38,39]. The PEL is modeled as a fixed charge region whose interface acts as a semipermeable membrane, allowing mobile electrolyte ions to exist both inside and outside the layer; this produces an electrostatic potential distribution that differs from an EDL near a bare surface [40,41]. Talebi et al. [42] examined hydrodynamic dispersion driven by a fully developed electroosmotic flow through soft microchannels coated with a dense PEL, using the Debye–Hückel linearization. Chen and Das [43] analyzed electroosmotic transport in a polyelectrolyte-grafted nanochannel with pH-dependent charge density. They showed that the PEL charge increases with its thickness, thereby enhancing the electrostatic potential and velocity at any transverse position. Sadeghi [38] investigated electroosmotic flow in soft microchannels and found that the mean velocity increases with the fixed charge density and PEL thickness but decreases with the friction coefficient arising from the same layer. Saha et al. [44] studied electroosmotic flow through a soft nanochannel coating the lower and upper channel walls with polyelectrolyte layers with similar or dissimilar physicochemical properties.

Additionally, several studies have modeled electroosmotic flow in channels whose charged hydrophobic supporting walls are grafted with an ion- and fluid-permeable PEL that traps a nonzero volumetric charge of positive or negative polarity. This configuration enables precise control over the interfacial slip and surface potential [45,46]. In this framework, Li and Jian [47] investigated solute dispersion using an alternating-current electroosmotic flow in a slit polyelectrolyte-grafted nanochannel with interfacial slip. Zheng and Jian [48] imposed a hydrodynamic slip condition in electroosmotic thrusters in soft nanochannels for space propulsion. Matin and Ohshima [49] and Zhao and Jian [50] analyzed mixed electroosmotic and pressure-driven flow through soft nanochannels, accounting for slip boundary conditions and a constant wall charge density.

Since the electrostatic potential on a PEL-coated surface can reach up to 300 mV [51,52], the modeling of electroosmotic flow must include steric effects when surface potentials exceed the Debye–Hückel limit [53,54]. The steric effect, which accounts for the excluded volume due to the finite ionic size, modifies the electric potential distribution through the steric factor $\nu =a^{3}n_{0}$, where *a* is the effective ionic size and $n_{0}$ is the ionic concentration of the bulk solution. This effect arises when a high voltage is applied to a surface in contact with an electrolyte, causing ions to crowd near the surface [55,56]. Therefore, the modified Poisson–Boltzmann equation should be used to capture the steric effect on the electric potential distribution in electroosmotic flow problems [54,57,58,59,60]. Xing and Jian [61] and Liu and Jian [62] developed numerical solutions for mixed pressure- and electroosmotically driven flow in soft nanochannels, accounting for the impact of steric effects on hydrodynamics and entropy generation, respectively. Seifollahi and Ashrafizadeh [63] conducted an analytical study of the influence of ionic size on electroosmotic flow through an ion-selective soft slit nanochannel. They found that the steric effects at high charge densities of the PELs dramatically alter the electric potential and flow. In recent investigations, Zheng et al. [64], Saha et al. [46], and Jimenez et al. [65] also analyzed how steric effects and other critical parameters influence electroosmotic flow in soft channels.

Although many fluids exhibit a nonlinear relationship between stress and strain rate, electroosmotic transport is typically studied with Newtonian fluids. As a result, numerous studies have focused on non-Newtonian fluids in electroosmotic flows to understand better the interplay among complex rheology, hydrodynamic and electrostatic phenomena, species transport, and heat transfer [66,67,68,69,70,71]. In accordance, Kumar and Barman [72] conducted a parametric study of electroosmotic flow and ion transport through a polyelectrolyte-grafted soft nanochannel using a power-law fluid. Their work highlighted how the rheological behavior and softness parameter of the polyelectrolyte influence the flow modulation and the selectivity of mobile electrolyte ions. Patel et al. [73,74] investigated the electroosmotic transport of biofluids in soft microchannels, assuming power-law, Bingham, Casson, and Herschel–Bulkley rheological models. Gaikwad et al. [75] studied electroosmotic flow in a soft nanochannel and reported the complex interplay between the non-Newtonian rheology of power-law fluids and the interfacial chemistry of the PEL on the walls.

On the other hand, transient analyses provide crucial insight into electrokinetic phenomena by revealing interactions between electrostatic forces and hydrodynamic effects that are not observable under steady-state conditions [76]. In this regard, Gandharv and Kaushik [77] studied the transient electroosmotic flow of a Newtonian liquid in a rotating soft microchannel. They found that the PEL thickness and its drag determine flow velocities and oscillations, which can be leveraged in lab-on-CD-based systems to control mass and momentum transport. Li et al. [78,79], Kumar and Jangili [80], and Shang et al. [81] derived analytical solutions for time-periodic electroosmotic flow in polyelectrolyte-grafted channels, considering Newtonian, Jeffrey, couple-stress and Maxwell fluids, alternating-current (AC) electric fields, the Debye–Hückel approximation, and no-slip boundary conditions. Sin et al. [82] conducted a semi-analytical study of the start-up electroosmotic flow of general Maxwell fluids through microchannels grafted with a multilayer of polyelectrolyte brushes under the Debye–Hückel approximation. They found that adjusting the conformation and charge distribution of polyelectrolyte brushes can control the flow direction.

As micro- and nanofluidics advance rapidly, understanding the fundamental mechanisms of fluid behavior at small scales becomes increasingly crucial [83]. In this direction, viscoelastic fluids, such as polymer solutions, blood, saliva, cerebrospinal fluid, droplets in emulsions, bubbles in foams, and particles in suspensions, exhibit complex flow dynamics in an electric field, requiring careful examination [84], including studies of electroosmotic flow at high surface potentials [85,86,87,88,89]. Also, because electroosmotic flow is increasingly important in research due to its applications in chemical engineering, biomedicine, nanotechnology, and process industries, further research on this flow in non-Newtonian fluids is essential to cover emerging applications and optimize the design and operation of small-scale devices [68,90,91,92,93,94,95]. With this motivation, and to the best of the authors’ knowledge, the start-up of electroosmotic flow of Maxwell fluids in soft channels at high surface potentials has not been examined. Therefore, by identifying this gap in the literature, this research conducts a parametric study on the interaction between the non-Newtonian rheology of fluids and electrostatic phenomena in the dynamics of electroosmotic flow. The mathematical model is based on the modified Poisson–Boltzmann and Cauchy momentum equations and is solved numerically using the commercial software COMSOL Multiphysics 6.1. The study discusses in detail the combined effects of PEL thickness, ionic concentration of the fixed charge, finite ionic size, dissimilar polarities of the PEL coating at the walls, drag resistance from the PEL, Maxwell fluid rheology, and hydrodynamic slip at the walls on the electroosmotic flow.

## 2. Problem Formulation

### 2.1. Physical Model

**Figure 1:**
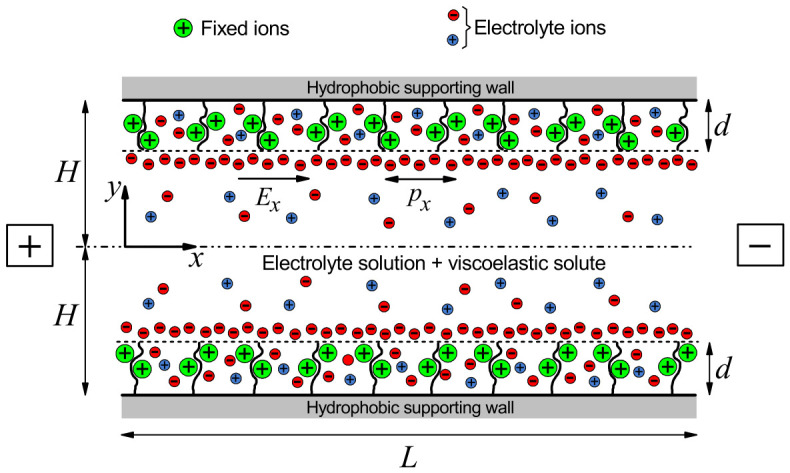
Sketch of electroosmotic flow of Maxwell fluids through a soft channel.

This research examines the transient electroosmotic flow of Maxwell fluids in a soft parallel-plate channel with charged, hydrophobic supporting walls coated by PELs containing fixed ions, as illustrated in Figure 1. The channel geometry is characterized by a plate spacing of 2H, a length *L*, and a PEL thickness *d* on each wall. The Cartesian coordinate system (x,y) is centered on the channel. The working fluid, a binary symmetric electrolyte mixed with a viscoelastic solute, fills the entire channel domain, including the permeable PELs. Consequently, bulk electrolyte ions are distributed both inside and outside the PELs. To generate electroosmotic flow, electrodes at the channel ends apply a constant electric field, $E_{x}$, in the *x*-direction, thereby inducing fluid motion. Although the primary driving force for fluid motion comes from electroosmotic effects, an external pressure gradient, $p_{x}=dp/dx$, may also be imposed. In this analysis, the concentration and polarity of fixed charges in the PELs can be either similar or different, leading to symmetric or asymmetric flow configurations. Hence, the PEL coatings, along with the charged and hydrophobic supporting walls, regulate both the electrostatic and hydrodynamic responses of the electroosmotic flow. Furthermore, steric effects are considered when determining the flow field, since surface charge control can achieve wall potentials exceeding 25 mV, and the electrolyte concentration can be increased to $10^{-2}$ M.

### 2.2. Governing Equations

The background electrolyte solution, which contains a viscoelastic solute, permeates the entire channel, filling both the electrolyte and PEL regions. Therefore, the continuity equation in both regions is given by:

(1) ∇·v=0,

where v is the velocity vector. To accurately determine the electroosmotic flow field, the presence of the PEL must be considered, as the governing equations for electrostatics and momentum differ from those applicable to classical rigid channel flows (without the PEL). Therefore, under the assumption of ionic equilibrium, the Poisson equations for the electric potential in the EDLs [38,41,79] and the modified Cauchy momentum equations [79,82,96,97] for both regions are described below. First, in the electrolyte layer:

(2) $$\nabla^{2}\psi =-\frac{\rho_{e}}{\varepsilon }$$

and

(3) $$\rho \frac{Dv}{Dt}=-\nabla p-\nabla \cdot \tau +\rho_{e}E,$$

and second, for the PEL:

(4) $$\nabla^{2}\psi =-\frac{\left(\rho_{e}+\rho_{PEL}\right)}{\varepsilon }$$

and

(5) $$\rho \frac{Dv}{Dt}=-\nabla p-\nabla \cdot \tau -kv+\rho_{e}E,$$

where, in Equations (2)–(5), ψ is the electric potential within the EDLs located at the channel system, $\rho_{e}=ez\left(n_{+}-n_{-}\right)$ is the volumetric free charge density from the electrolyte, *e* is the elementary charge, *z* is the absolute value of the valences of a symmetric (z:z) electrolyte with $z=z_{+}=z_{-}$, $z_{+}$ and $z_{-}$ are the valence of the positive and negative ions, $n_{+}$ and $n_{-}$ are the number concentrations of the positive and negative ions, ε is the dielectric permittivity, ρ is the fluid density, *t* is the time, *p* is the pressure, τ is the stress tensor, E is the electric field vector, $\rho_{PEL}=ZeN$ is the volumetric fixed charge density from the PEL, *Z* and *N* are the valence and the ionic number concentration of the PEL ions, and *k* is the friction coefficient. Equation (4) incorporates both fixed charges and electrolyte ions to control the electric potential distribution, thus reflecting the permeable nature of the PEL through the Poisson equation. Meanwhile, the third term on the right-hand side of Equation (5) represents a friction force generated by the porous matrix of the PEL, attributed to the presence of polyelectrolyte grafts. This friction force is well-known as the Darcy drag force. It is important to note that the fixed ions on the polyelectrolyte brushes do not contribute to fluid motion, since they are immobile; therefore, the body force exerted on the fluid and represented by the fourth term on the right-hand side of Equation (5) only includes the free charge density from the electrolyte.

The rheological behavior of the viscoelastic fluid follows the linear Maxwell model given below [69,82,97,98,99,100]:

(6) $$\tau +\lambda \frac{\partial \tau }{\partial t}=-\eta_{0}\dot{\gamma },$$

where λ is the relaxation time, $\eta_{0}$ is the zero-shear-rate viscosity, $\dot{\gamma }=\left(\nabla v\right)+{\left(\nabla v\right)}^{T}$ is the rate-of-strain tensor, and ${\left(\nabla v\right)}^{T}$ is the transpose of the tensor (∇v).

In addition, since high surface potentials and high electrolyte ionic concentrations are considered in this investigation, and under the mean-field description of steric effects in equilibrium for a symmetric (z:z) electrolyte, the modified Boltzmann distribution accounting for ionic size is established as follows [55,61,62,64]:

(7) $$n_{\pm }=\frac{n_{0}}{1+2\nu \left[\cosh\left(\frac{ze\psi }{k_{B}T}\right)-1\right]}\exp\left(\mp \frac{ze\psi }{k_{B}T}\right),$$

where $n_{0}$ is the ionic number concentration in the bulk solution, $\nu =a^{3}n_{0}$ is the steric factor, *a* is the effective ionic size, $k_{B}$ is the Boltzmann constant, and *T* is the temperature. In Equation (7), the term $1+2\nu \left[\cosh\left(\frac{ze\psi }{k_{B}T}\right)-1\right]$ can be defined as [41,55,61,62,64]:

(8) $$1+2\nu \left[\cosh\left(\frac{ze\psi }{k_{B}T}\right)-1\right]=1+4\nu \sinh^{2}\left(\frac{ze\psi }{2k_{B}T}\right)=1-2\nu +2\nu \cosh\left(\frac{ze\psi }{k_{B}T}\right).$$

Taking into account the following assumptions, the electroosmotic flow can be considered unidirectional on the *x*-axis:

- Laminar and fully developed flow [42,50,61,62,75];
- Transient flow [79,82];
- Channel length *L* much greater than the channel height 2H [61,75,79];
- Flow driven by the combined effects of the electroosmotic transport along with an imposed constant pressure gradient [40,49,50,75];
- Constant physical properties [40,62,75];
- Electroosmotic flow with insignificant or no overlap effect of the EDLs [50,75,101,102].

Therefore, under these assumptions, substituting Equations (6) and (7) into Equations (2)–(5) yields the following simplified forms of the Poisson–Boltzmann and momentum equations for the two regions in the channel. First, the governing equations for the electrolyte layer at (−H+d)<y<(H−d) are:

(9) $$\frac{\partial^{2}\psi \left(y\right)}{\partial y^{2}}=\frac{2zen_{0}}{\varepsilon }\frac{\sinh\left(\frac{ze\psi (y)}{k_{B}T}\right)}{1+2\nu \left[\cosh\left(\frac{ze\psi (y)}{k_{B}T}\right)-1\right]}$$

and

(10) $$\rho \frac{\partial u(y,t)}{\partial t}=-\frac{dp}{dx}-\frac{\partial \tau_{yx}\left(y,t\right)}{\partial y}-\frac{2zen_{0}\sinh\left(\frac{ze\psi (y)}{k_{B}T}\right)}{1+2\nu \left[\cosh\left(\frac{ze\psi (y)}{k_{B}T}\right)-1\right]}E_{x},$$

and second, the governing equations for the PEL regions at −H<y<(−H+d) and (H−d)<y<H are:

(11) $$\frac{\partial^{2}\psi \left(y\right)}{\partial y^{2}}=\frac{2zen_{0}}{\varepsilon }\frac{\sinh\left(\frac{ze\psi (y)}{k_{B}T}\right)}{1+2\nu \left[\cosh\left(\frac{ze\psi (y)}{k_{B}T}\right)-1\right]}-\frac{Z_{b,t}eN_{b,t}}{\varepsilon }$$

and

(12) $$\rho \frac{\partial u(y,t)}{\partial t}=-\frac{dp}{dx}-\frac{\partial \tau_{yx}\left(y,t\right)}{\partial y}-ku\left(y,t\right)-\frac{2zen_{0}\sinh\left(\frac{ze\psi (y)}{k_{B}T}\right)}{1+2\nu \left[\cosh\left(\frac{ze\psi (y)}{k_{B}T}\right)-1\right]}E_{x},$$

where in Equations (10) and (12), *u* denotes the axial velocity and $\tau_{yx}$ represents the shear stress given by the following expression:

(13) $$\left(1+\lambda \frac{\partial }{\partial t}\right)\tau_{yx}\left(y,t\right)=-\eta_{0}\frac{\partial u(y,t)}{\partial y}.$$

In addition, the subscripts in Equation (11) denote the PEL properties at the bottom (“b”) and top (“t”) walls.

To solve the mathematical model of electroosmotic flow, initial and boundary conditions are required for Equations (9)–(13). Thus, the initial conditions for the entire flow domain at −H≤y≤H indicate that both velocity, shear stress, and acceleration are equal to zero, reflecting a state of rest, as follows:

(14) $${\left.u\right|}_{\left(y,t=0\right)}=0,{\left.\tau_{yx}\right|}_{\left(y,t=0\right)}=0,{\left.\frac{\partial u}{\partial t}\right|}_{\left(y,t=0\right)}=0.$$

Concerning the boundary conditions for t>0, firstly consider at the bottom (y=−H) and top (y=+H) walls of the channel the Gauss [48,49,50,61,64,103] and the hydrodynamic Navier-slip [100,104,105] boundary conditions into the PEL regions, which are indicated as follows:

(15) $${\left.\frac{d\psi }{dy}\right|}_{\left(y=\mp H\right)}=\mp \frac{\sigma }{\varepsilon },\;{\left.u\right|}_{\left(y=\mp H,t\right)}=\pm \frac{b}{\eta_{0}}{\left.\tau_{yx}\right|}_{\left(y=\mp H,t\right)},$$

where σ is the surface charge density of the bare wall, and *b* is the slip length. The signs above the terms “∓σ/ε” and “$\pm b\tau_{yx}/\eta_{0}$” correspond to the bottom wall, whereas the signs below correspond to the top wall, respectively. Moreover, in Equation (15), the second boundary condition accounts for the hydrophobic character of the supporting walls of the channel.

Secondly, the boundary conditions at the interfaces between the electrolyte layer (EL) and the PEL near the bottom wall at y=(−H+d) and near the top wall at y=(+H−d) are the continuity of the electric potential, electric displacement, velocity continuity, and stress balance:

(16) $$\begin{array}{c} {\left[{\left.\psi \right|}_{\left(y=\mp H\pm d\right)}\right]}_{EL}={\left[{\left.\psi \right|}_{\left(y=\mp H\pm d\right)}\right]}_{PEL};\;{\left[{\left.\varepsilon \frac{d\psi }{dy}\right|}_{\left(y=\mp H\pm d\right)}\right]}_{EL}={\left[{\left.\varepsilon \frac{d\psi }{dy}\right|}_{\left(y=\mp H\pm d\right)}\right]}_{PEL}\\ {\left[{\left.u\right|}_{\left(y=\mp H\pm d,t\right)}\right]}_{EL}={\left[{\left.u\right|}_{\left(y=\mp H\pm d,t\right)}\right]}_{PEL};\;{\left[{\left.\tau_{yx}\right|}_{\left(y=\mp H\pm d,t\right)}\right]}_{EL}={\left[{\left.\tau_{yx}\right|}_{\left(y=\mp H\pm d,t\right)}\right]}_{PEL}, \end{array}$$

where the application positions are denoted as y=(∓H±d), with the signs above corresponding to the EL/PEL interface near the bottom wall, and the signs below corresponding to the EL/PEL interface near the top wall, respectively.

### 2.3. Dimensionless Mathematical Model

The mathematical model presented in the previous section is normalized by introducing the following dimensionless variables:

(17) $$\bar{y}=\frac{y}{H},\;\bar{t}=\frac{\eta_{0}t}{\rho H^{2}},\;\bar{\psi }\left(\bar{y}\right)=\frac{ze\psi (y)}{k_{B}T},\;\bar{u}\left(\bar{y},\bar{t}\right)=\frac{u(y,t)}{u_{c}},\;{\bar{\tau }}_{yx}\left(\bar{y},\bar{t}\right)=\frac{H\tau_{yx}\left(y,t\right)}{\eta_{0}u_{c}},$$

where the characteristic velocity is $u_{c}=-\varepsilon k_{B}TE_{x}/ze\eta_{0}$ [38,42,62,65]. Therefore, the governing and constitutive Equations (9)–(13) are rewritten as follows. In the case of the electrolyte region at $\left(-1+\bar{d}\right)<\bar{y}<\left(1-\bar{d}\right)$, the Poisson–Boltzmann and momentum equations are represented in dimensionless form as:

(18) $$\frac{\partial^{2}\bar{\psi }\left(\bar{y}\right)}{\partial {\bar{y}}^{2}}={\bar{\kappa }}^{2}\frac{\sinh\left(\bar{\psi }\left(\bar{y}\right)\right)}{1+2\nu \left[\cosh\left(\bar{\psi }\left(\bar{y}\right)\right)-1\right]}$$

and

(19) $$\frac{\partial \bar{u}\left(\bar{y},\bar{t}\right)}{\partial \bar{t}}=\Gamma -\frac{\partial {\bar{\tau }}_{yx}\left(\bar{y},\bar{t}\right)}{\partial \bar{y}}+{\bar{\kappa }}^{2}\frac{\sinh\left(\bar{\psi }\left(\bar{y}\right)\right)}{1+2\nu \left[\cosh\left(\bar{\psi }\left(\bar{y}\right)\right)-1\right]},$$

and in the case of the PEL regions at $-1<\bar{y}<\left(-1+\bar{d}\right)$ and $\left(1-\bar{d}\right)<\bar{y}<1$:

(20) $$\frac{\partial^{2}\bar{\psi }\left(\bar{y}\right)}{\partial {\bar{y}}^{2}}={\bar{\kappa }}^{2}\frac{\sinh\left(\bar{\psi }\left(\bar{y}\right)\right)}{1+2\nu \left[\cosh\left(\bar{\psi }\left(\bar{y}\right)\right)-1\right]}-\mathrm{sgn}\left(Z_{b,t}\right){\bar{\kappa }}_{PEL;b,t}^{2}$$

and

(21) $$\frac{\partial \bar{u}\left(\bar{y},\bar{t}\right)}{\partial \bar{t}}=\Gamma -\frac{\partial {\bar{\tau }}_{yx}\left(\bar{y},\bar{t}\right)}{\partial \bar{y}}-\alpha^{2}\bar{u}\left(\bar{y},\bar{t}\right)+{\bar{\kappa }}^{2}\frac{\sinh\left(\bar{\psi }\left(\bar{y}\right)\right)}{1+2\nu \left[\cosh\left(\bar{\psi }\left(\bar{y}\right)\right)-1\right]}.$$

The dimensionless parameters arising in Equations (18)–(21) are defined as follows:

(22) $$\bar{\kappa }=\frac{H}{\kappa^{-1}},\;{\bar{\kappa }}_{PEL;b,t}=\frac{H}{\kappa_{PEL;b,t}^{-1}},\;\Gamma =-\frac{H^{2}p_{x}}{\eta_{0}u_{c}},\;\alpha =H\sqrt{\frac{k}{\eta_{0}}},\;\bar{d}=\frac{d}{H},$$

where $\bar{\kappa }$ is the electrokinetic parameter of the electrolyte, defined as the ratio of half the channel height to the Debye length in the EDL defined as $\kappa^{-1}={\left[\left(\varepsilon k_{B}T\right)/\left(2e^{2}z^{2}n_{0}\right)\right]}^{1/2}$ and also known as EDL thickness. ${\bar{\kappa }}_{PEL;b,t}$ or separately, ${\bar{\kappa }}_{PEL,b}$ and ${\bar{\kappa }}_{PEL,t}$, are the electrokinetic parameters of the PEL at the bottom (“*b*”) and top (“*t*”) walls, respectively, and are defined as the ratio of half the channel height to the equivalent Debye length of the PEL defined as $\kappa_{PEL;b,t}^{-1}={\left[\left(\varepsilon k_{B}T\right)/(ze^{2}|Z_{b,t}\left|N_{b,t}\right)\right]}^{1/2}$ and also known as equivalent EDL thicknesses. Γ is the dimensionless pressure gradient, defined as the ratio of pressure forces to electroosmotic forces. α is the drag parameter, defined as the ratio of the resisting forces generated by the PEL brushes to viscous forces in the system, and quantifies the impact of the Darcy drag in the PEL. $\bar{d}$ is the dimensionless PEL thickness, defined as the ratio of the PEL thickness to half the channel height. Finally, the sign function was introduced in Equation (20) to control the polarity of the fixed charges in the PELs, where $\mathrm{sgn}(Z_{b,t})=1$ for $Z_{b,t}>0$ and $\mathrm{sgn}(Z_{b,t})=-1$ for $Z_{b,t}<0$. Taking into account the above, this work uses the parameters ${\bar{\kappa }}_{PEL,b}$ and ${\bar{\kappa }}_{PEL,t}$ and the sign function ($\mathrm{sgn}(Z_{b,t})$) to explore the variability of the ionic concentration of fixed charges ($N_{b}$ and $N_{t}$) and the change in polarity of the valency of fixed ions ($Z_{b,t}$) in the PELs, to promote symmetric and asymmetric flows under corresponding conditions of symmetric and asymmetric surface charge distributions at the channel walls.

Regarding the rheological Maxwell model, replacing the dimensionless variables of Equation (17) into Equation (13) yields:

(23) $$\left(1+\bar{\lambda }\frac{\partial }{\partial \bar{t}}\right){\bar{\tau }}_{yx}\left(\bar{y},\bar{t}\right)=-\frac{\partial \bar{u}\left(\bar{y},\bar{t}\right)}{\partial \bar{y}},$$

with

(24) $$\bar{\lambda }=\frac{\eta_{0}\lambda }{\rho H^{2}}$$

where $\bar{\lambda }$ is the dimensionless relaxation time and quantifies the competition between elastic and viscous effects in the fluid. This parameter is defined as the ratio of the relaxation time to the characteristic time for the deformation process.

In this context, the initial and boundary conditions for Equations (18)–(21) and (23) are obtained in dimensionless form by substituting Equation (17) into Equations (14)–(16). Then, for the entire dimensionless geometrical domain of the channel $-1\leq \bar{y}\leq 1$, the initial conditions at $\bar{t}=0$ from Equation (14) are:

(25) $${\left.\bar{u}\right|}_{\left(\bar{y},\bar{t}=0\right)}=0,{\left.{\bar{\tau }}_{yx}\right|}_{\left(\bar{y},\bar{t}=0\right)}=0,{\left.\frac{\partial \bar{u}}{\partial \bar{t}}\right|}_{\left(\bar{y},\bar{t}=0\right)}=0.$$

The dimensionless boundary conditions of Gauss and hydrodynamic slip for $\bar{t}>0$ at the bottom and top walls from Equation (15) are respectively:

(26) $${\left.\frac{d\bar{\psi }}{d\bar{y}}\right|}_{\left(\bar{y}=\mp 1\right)}=\mp \Omega ,\;{\left.\bar{u}\right|}_{\left(\bar{y}=\mp 1,\bar{t}\right)}=\pm \bar{b}{\left.{\bar{\tau }}_{yx}\right|}_{\left(\bar{y}=\mp 1,\bar{t}\right)},$$

with

(27) $$\Omega =\frac{\sigma ezH}{\varepsilon k_{B}T},\;\bar{b}=\frac{b}{H},$$

where Ω is the dimensionless surface charge density and characterizes the polarization strength of the bare channel walls. $\bar{b}$ is the dimensionless hydrodynamic slip length, defined as the ratio of the slip length to half the channel height, which controls the hydrophobic effect at the channel walls.

Additionally, the dimensionless boundary conditions for the continuity of the electric potential, electric displacement, velocity continuity, and stress balance for the interfaces between the electrolyte layer and the PELs are obtained from Equation (16) and can be written as:

(28) $$\begin{array}{c} {\left[{\left.\bar{\psi }\right|}_{\left(\bar{y}=\mp 1\pm \bar{d}\right)}\right]}_{EL}={\left[{\left.\bar{\psi }\right|}_{\left(\bar{y}=\mp 1\pm \bar{d}\right)}\right]}_{PEL};\;{\left[{\left.\frac{d\bar{\psi }}{d\bar{y}}\right|}_{\left(\bar{y}=\mp 1\pm \bar{d}\right)}\right]}_{EL}={\left[{\left.\frac{d\bar{\psi }}{d\bar{y}}\right|}_{\left(\bar{y}=\mp 1\pm \bar{d}\right)}\right]}_{PEL}\\ {\left[{\left.\bar{u}\right|}_{\left(\bar{y}=\mp 1\pm \bar{d},\bar{t}\right)}\right]}_{EL}={\left[{\left.\bar{u}\right|}_{\left(\bar{y}=\mp 1\pm \bar{d},\bar{t}\right)}\right]}_{PEL};\;{\left[{\left.{\bar{\tau }}_{yx}\right|}_{\left(\bar{y}=\mp 1\pm \bar{d},\bar{t}\right)}\right]}_{EL}={\left[{\left.{\bar{\tau }}_{yx}\right|}_{\left(\bar{y}=\mp 1\pm \bar{d},\bar{t}\right)}\right]}_{PEL}. \end{array}$$

To express the governing Equations (19) and (21), and the hydrodynamic slip and stress balance boundary conditions (within Equations (26) and (28)) in terms of velocity, these equations are multiplied by the operator $\left(1+\bar{\lambda }\frac{\partial }{\partial \bar{t}}\right)$ and by using Equation (23), yielding the following momentum equations for the electrolyte layer at $\left(-1+\bar{d}\right)<\bar{y}<\left(1-\bar{d}\right)$:

(29) $$\bar{\lambda }\frac{\partial^{2}\bar{u}\left(\bar{y},\bar{t}\right)}{\partial {\bar{t}}^{2}}+\frac{\partial \bar{u}\left(\bar{y},\bar{t}\right)}{\partial \bar{t}}=\Gamma +\frac{\partial^{2}\bar{u}\left(\bar{y},\bar{t}\right)}{\partial {\bar{y}}^{2}}+{\bar{\kappa }}^{2}\frac{\sinh\left(\bar{\psi }\left(\bar{y}\right)\right)}{1+2\nu \left[\cosh\left(\bar{\psi }\left(\bar{y}\right)\right)-1\right]},$$

and for the PEL regions at $-1<\bar{y}<\left(-1+\bar{d}\right)$ and $\left(1-\bar{d}\right)<\bar{y}<1$:

(30) $$\begin{array}{c} \bar{\lambda }\frac{\partial^{2}\bar{u}\left(\bar{y},\bar{t}\right)}{\partial {\bar{t}}^{2}}+\left(1+\alpha^{2}\bar{\lambda }\right)\frac{\partial \bar{u}\left(\bar{y},\bar{t}\right)}{\partial \bar{t}}=\\ \Gamma +\frac{\partial^{2}\bar{u}\left(\bar{y},\bar{t}\right)}{\partial {\bar{y}}^{2}}-\alpha^{2}\bar{u}\left(\bar{y},\bar{t}\right)+{\bar{\kappa }}^{2}\frac{\sinh\left(\bar{\psi }\left(\bar{y}\right)\right)}{1+2\nu \left[\cosh\left(\bar{\psi }\left(\bar{y}\right)\right)-1\right]}, \end{array}$$

Also, the hydrodynamic slip boundary conditions at the bottom and top walls result in:

(31) $${\left.\bar{u}\right|}_{\left(\bar{y}=\mp 1,\bar{t}\right)}+\bar{\lambda }{\left.\frac{\partial \bar{u}}{\partial \bar{t}}\right|}_{\left(\bar{y}=\mp 1,\bar{t}\right)}=\pm \bar{b}{\left.\frac{\partial \bar{u}}{\partial \bar{y}}\right|}_{\left(\bar{y}=\mp 1,\bar{t}\right)},$$

and correspondingly, the stress balance at the interfaces between the electrolyte layer and the PELs is:

(32) $${\left[{\left.\frac{\partial \bar{u}}{\partial \bar{y}}\right|}_{\left(\bar{y}=\mp 1\pm \bar{d},\bar{t}\right)}\right]}_{EL}={\left[{\left.\frac{\partial \bar{u}}{\partial \bar{y}}\right|}_{\left(\bar{y}=\mp 1\pm \bar{d},\bar{t}\right)}\right]}_{PEL}.$$

## 3. Solution Methodology

**Figure 2:**
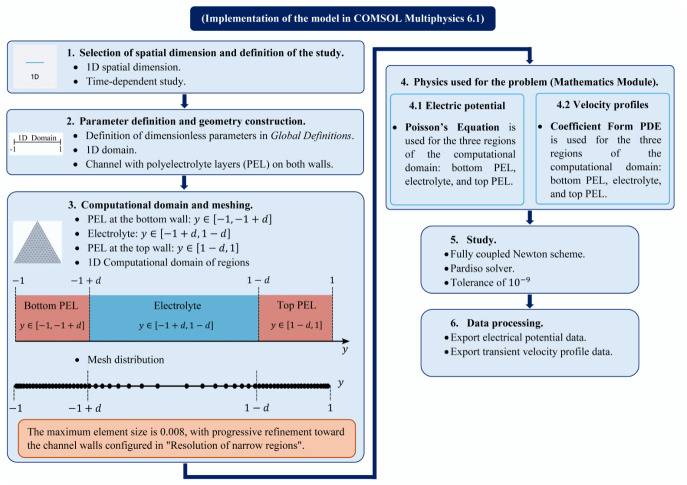
Diagram of the setup and the mesh structure in COMSOL Multiphysics 6.1.

The dimensionless nonlinear equations that describe the electroosmotic flow of Maxwell fluids in a polyelectrolyte-coated channel are numerically solved using the commercial software COMSOL Multiphysics 6.1 in the *Mathematics* module. A schematic representation of the numerical implementation is shown in Figure 2, where the one-dimensional computational domain that represents the channel transverse coordinate $\bar{y}$∈[−1,1] is partitioned into three subdomains corresponding to the bottom PEL region, bulk electrolyte region, and top PEL region. The electric potential $\bar{\psi }\left(\bar{y}\right)$ is obtained in a steady-state analysis by solving Equations (18) and (20) with their corresponding boundary conditions in Equations (26) and (28), and is solved using the *Poisson’s Equation* interface from the *Classical PDEs* branch in each subdomain. On the other hand, the velocity field $\bar{u}\left(\bar{y},\bar{t}\right)$ coupled with the electric potential solution is calculated in a transient analysis by solving Equations (29) and (30) with their respective initial and boundary conditions in Equations (25), (28), (31) and (32), using the *Coefficient Form PDE* interface in the corresponding subdomains. The transient analysis is conducted with a constant time step of $\Delta \bar{t}=0.01$, which provides sufficient temporal resolution to capture the dynamics of the Maxwell fluid. Here, the slip conditions on the walls are imposed with a weak contribution on the boundaries. The domain is discretized using the finite element method with quadratic Lagrange elements and an automatic mesh refinement strategy. Thus, the maximum size of the elements is set at the channel center, while a gradual reduction in element size is applied toward the channel walls, with a resolution of 3 in the narrow regions of the PEL. Therefore, the mesh density gradually increases towards the PEL regions, where the highest gradients of $\bar{\psi }\left(\bar{y}\right)$ and $\bar{u}\left(\bar{y},\bar{t}\right)$ occur, ensuring an accurate spatial resolution for the EDLs and for the velocity boundary effects.

**Figure 3:**
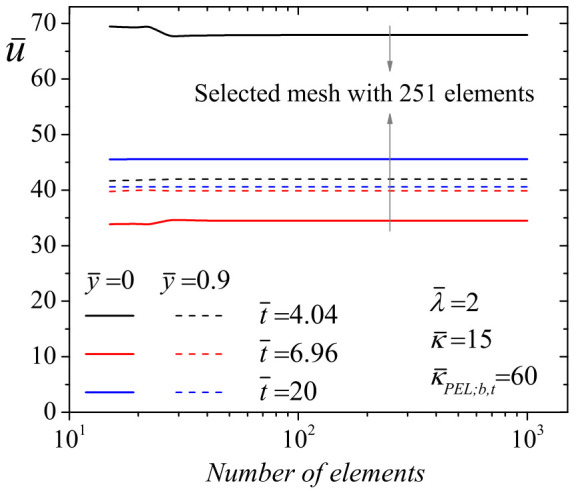
Mesh independence study on the electroosmotic flow with $\mathrm{sgn}(Z_{b,t})=1$, $\bar{b}=0.05$, $\bar{d}=0.1$, α=1, Γ=1, Ω=5, and ν=0.04.

To obtain reliable results, a mesh independence study was performed for the electroosmotic flow, with the parameters shown in Figure 3. Here, the dimensionless velocity is shown at two positions of the transverse coordinate $\bar{y}$ and at three different times, where the first two times at $\bar{t}=4.04$ and 6.96 are in the transient stage, and the last value at $\bar{t}=20$ represents an arbitrary time within the steady-state regime. The results were obtained considering different maximum element sizes, ranging from 0.4 to 0.002, which generate 15 and 1000 elements, respectively. This study finds that when the mesh size is 0.008 or smaller, which generates 251 elements, the velocity remains constant and meets the convergence criterion $\varepsilon_{s}=0.001\%$. This result was obtained using the relative error $\left|\varepsilon_{a,i}\right|=\left|\left({\bar{u}}_{i}-{\bar{u}}_{j}\right)/{\bar{u}}_{i}\right|\cdot 100\%\leq \varepsilon_{s}$, where ${\bar{u}}_{i}$ and ${\bar{u}}_{j}$ are the current and previous values of the velocity $\bar{u}\left(\bar{y},\bar{t}\right)$ in a mesh with a specific number of elements. Table 1 presents a summary of the velocity values and their respective relative errors $\varepsilon_{a,i}$ for different numbers of elements and considering two representative cases from Figure 3. Additionally, the discrete system was solved using a *fully coupled Newton* scheme with automatic damping and the *PARDISO* direct solver. Finally, the solver tolerance was set to $10^{-9}$, ensuring stability, accuracy, and reproducibility without requiring additional numerical stabilization.

## 4. Results

This section analyzes the numerical results for the transient electroosmotic flow of a viscoelastic Maxwell fluid under an external direct current (DC) electric field. The simulations are performed using ranges of physical parameters reported in the literature: 0.05≤H≤10μm [39,42,54,63,82]; 0<d≤198 nm [39,42,46,51]; 0≤b≤180 nm [45,46,47,48,58]; 0≤a≤ 7 nm [54,55,60,63]; $\eta_{0}=10^{-3}$ Pa s, ρ=1000 kg $m^{-3}$, and $\varepsilon =7\times 10^{-10}$ C $V^{-1}m^{-1}$ [69,77,79]; $z_{+}=z_{-}=z=1$ [25,39,63]; $1\leq \kappa^{-1}\leq 300$ nm [25,42,48,74], approximately for $10^{-6}\leq M\leq 10^{-1}$ mol $L^{-1}$ (or $6.022\times 10^{20}\leq n_{0}\leq 6.022\times 10^{25}m^{-3}$, where for a symmetric electrolyte, $\kappa^{-1}=\left(3.04\times 10^{-10}\right)/\left(z\sqrt{M}\right)$ [25]), respectively; $2.4\times 10^{23}\leq N\leq 1.20\times 10^{26}m^{-3}$ [39,42,45,48,63]; Z=±1 [39,44,48,49]; $3.30\times 10^{11}\leq k\leq 2.87\times 10^{17}$ Pa s $m^{-2}$ (based on the square root of the ratio of viscosity and friction coefficient $0.059\leq {\left(\eta_{0}/k\right)}^{1/2}\leq 55$ nm and by considering equal viscosities for the PEL and the electrolyte layer) [39,42,63]; 0≤dp/dx≤2250 kPa $m^{-1}$ (based on Δp/L with a pressure difference of Δp=45 kPa and a channel length of L=0.02 m) [106]; $0\leq \lambda \leq 10^{-4}$ s [69,107]; $E_{x}\leq 1000$ V $m^{-1}$ [39,42,44,69]; $0\leq \sigma \leq 5.4\times 10^{-3}$ C $m^{-2}$ [46,64,82]. In this regard, the values of the constants are: T=298.15 K, $k_{B}=1.381\times 10^{-23}$ K $J^{-1}$, and $e=1.602\times 10^{-19}$ C. Therefore, an appropriate combination of these physical parameters defines the range of the dimensionless parameters in the mathematical model governing the electroosmotic flow, as follows: $0<\bar{d}\leq 0.2$, $4\leq \bar{\kappa }\leq 15$, $5\leq {\bar{\kappa }}_{PEL}\leq 60$, 0<Ω≤50, $0<\bar{b}\leq 0.05$, 0≤α≤7, 0≤ν≤0.04, $0<\bar{\lambda }\leq 5$, and 0≤Γ≤10.

### 4.1. Validation

**Figure 4:**
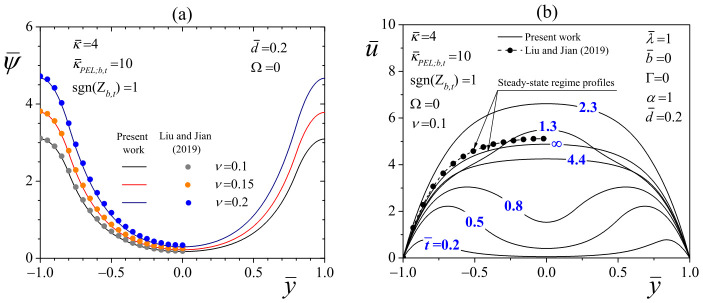
Validation of the (**a**) electric potential distribution and (**b**) electroosmotic flow velocity of the present work with the research reported by Liu and Jian [62] in dimensionless form.

To validate the numerical results presented in this work against previous studies, Figure 4 compares the electric potential distribution and velocity profiles of the electroosmotic flow in a polyelectrolyte-coated flat-plate channel from Liu and Jian [62] with those obtained in the present study. The dimensionless parameters used to model the electroosmotic flow are presented in the validation plots, where the equivalence of the electrokinetic parameters in both studies is given by $\bar{\kappa }=\lambda^{-1}=4$ and ${\bar{\kappa }}_{PEL}=\lambda_{FCL}^{-1}=10$ for the PEL region. In this context, Figure 4a compares the electric potential distribution where the solid lines correspond to the results of the present work, while the distributions with symbols represent those obtained by Liu and Jian [62]. As a result, this comparison demonstrates strong agreement between the two studies for all steric factor values. On the other hand, the solid lines in Figure 4b show the transient evolution of the velocity profiles with $\bar{\lambda }=1$, where $\bar{t}=\infty$ corresponds to the time at which the fluid reaches the steady-state and exhibits Newtonian flow behavior, equivalent to the profile obtained by Liu and Jian [62] in steady-state. This comparison reveals a slight discrepancy in the velocity magnitudes, which is attributed to the methodology employed by Liu and Jian [62] (the finite difference method), whereas the present work uses the finite element method in COMSOL. However, the velocity profiles show adequate qualitative agreement. To obtain quantitative comparisons in this validation, the Root Mean Square Error (RMSE) is estimated using the following expression:

(33) $$\mathrm{RMSE}\left(\chi \right)={\left[\frac{\sum_{q=1}^{m}{\left(\chi_{a,q}-\chi_{b,q}\right)}^{2}}{\sum_{q=1}^{m}\chi_{a,q}^{2}}\right]}^{1/2}\times 100,$$

where $\chi \in \{\bar{u},\;\bar{\psi }\}$ takes the values of the electric potential or velocity, as appropriate; $\chi_{a,q}$ are the parameter values from the present numerical results; $\chi_{b,q}$ are the values from the comparison results (from Liu and Jian [62]) at each node *q*; and m=26 is the number of data considered. Thus, the RMSE values for the electric potential in Figure 4a are 2.32%, 2.47%, and 2.69% for ν=0.1, ν=0.15, and ν=0.2, respectively. For the velocity case in Figure 4b, an RMSE value of 4.9% is obtained. Thus, the numerical results obtained in this study are consistent with those reported by Liu and Jian [62], indicating that the numerical solution adequately describes the behavior of the electroosmotic flow.

### 4.2. Electric Potential Distribution

**Figure 5:**
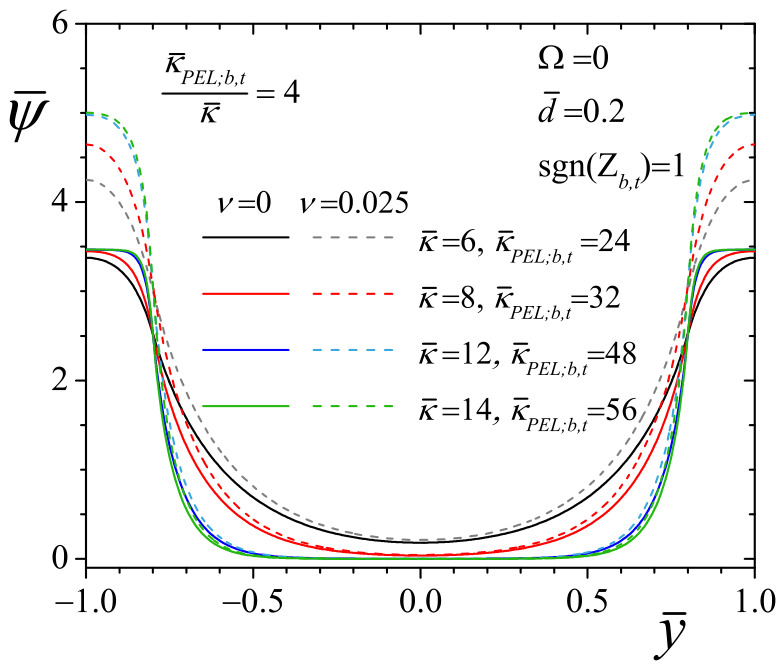
Dimensionless electric potential distribution as a function of the dimensionless transverse coordinate $\bar{y}$ and the increment of the electrokinetic parameters $\bar{\kappa }$ and ${\bar{\kappa }}_{PEL}$ while maintaining a constant ratio of ${\bar{\kappa }}_{PEL}/\bar{\kappa }=4$, with two steric factor values ν(=0,0.025) and the symmetric case of $\mathrm{sgn}(Z_{b,t})=1$.

Figure 5 shows the electric potential distribution $\bar{\psi }$ as a function of the dimensionless transverse coordinate $\bar{y}$, highlighting the influence of the electrokinetic parameters $\bar{\kappa }$ and ${\bar{\kappa }}_{PEL}$, as well as the steric factor ν. The other parameters, including the PEL thickness $\bar{d}=0.2$, the surface charge density Ω=0, and the polarity of the fixed charges $\mathrm{sgn}(Z_{b,t})=1$, are kept constant. The ranges of the electrokinetic parameters are $6\leq \bar{\kappa }\leq 14$ and $24\leq {\bar{\kappa }}_{PEL;b,t}\leq 56$, with the combinations shown in the figure for a fixed ratio of ${\bar{\kappa }}_{PEL;b,t}/\bar{\kappa }=4$. From this figure, it can be observed that in the absence of the steric effect, with ν=0, the electric potential near the walls shows a slight increase and approaches a flat region with a constant value as $\bar{\kappa }$ and ${\bar{\kappa }}_{PEL}$ increase. This behavior is associated with the formation of the Donnan potential, which occurs when the PEL thickness significantly exceeds the screening Debye length [108], that is, when $\bar{d}\gg 1/\bar{\kappa }$ (in physical form as $d\gg \kappa^{-1}$). In this scenario, mobile counterions within the layer help compensate for the fixed charges of the polyelectrolyte. This compensation establishes a balance between the free charge density and the fixed charge density within the PEL, leading to a condition of local electroneutrality (LEN) in the inner region, where $ez(n_{+}-n_{-})+ZeN=0$ [41]. This mechanism, also known as electrostatic screening, occurs when mobile counterions redistribute to neutralize the fixed charges and attenuate the local electric field. Consequently, the local electric field within the PEL tends to vanish, and the electric potential reaches a constant value, resulting in a plateau corresponding to the Donnan potential. Therefore, when ν=0, this condition begins to be fulfilled for $\bar{\kappa }=12$ and becomes clearly evident for $\bar{\kappa }=14$, where the condition $\bar{d}\gg 1/\bar{\kappa }$ is satisfied. In this regard, the analytical expression derived by Chanda and Das [41] for the Donnan potential, ${\bar{\psi }}_{D}=\ln\left\{\left[\left(1-2\nu \right)+\sqrt{{\left(1-2\nu \right)}^{2}+\left(K_{\lambda }^{4}-4\nu^{2}\right)}\right]/\left(K_{\lambda }^{2}-2\nu \right)\right\}$, predicts ${\bar{\psi }}_{D}=3.46$ for ν=0 and $K_{\lambda }=\bar{\kappa }/{\bar{\kappa }}_{PEL}=1/4$, which matches the plateau observed numerically; hence, the flat region for the potential distribution corresponds to the establishment of Donnan equilibrium within the PEL. On the other hand, the magnitude of the electric potential inside the PEL increases notably when ν=0.025. The finite ionic size limits the accumulation of counterions near the fixed charges, reducing the efficiency of electrostatic screening. As a result, the attainable ionic concentration is insufficient to fully compensate for the fixed charge and suppress the local electric field, thereby preventing the formation of a well-defined Donnan plateau. Therefore, a higher potential difference is required to mitigate the imbalance between the mobile and fixed charges within the PEL. Thus, this requirement for a higher surface potential in the presence of the steric effect with ν=0.025 shows an increasing trend as $\bar{\kappa }$ increases from $\bar{\kappa }=6$ to $\bar{\kappa }=14$, with the latter value corresponding to the condition in which the Donnan potential is approximated. This prediction of the increase in ${\bar{\psi }}_{D}$ as ν increases is consistent with the analytical expression of Chanda and Das [41] above; thus, evaluating for ν=0.025 and $K_{\lambda }=1/4$ yields ${\bar{\psi }}_{D}=5.02$, which agrees with the value of the wall potential observed in the dashed green line with ${\bar{\psi }}_{\nu =0.025}=5.0$. As can be seen, reaching the Donnan potential plateau in the presence of steric effects strictly requires that $\bar{d}\gg 1/\bar{\kappa }$. Therefore, following the case with ν=0.025 and $\bar{\kappa }=14$ (dashed green line), and although the PEL is approximately 2.8 times thicker than the Debye length, the potential at the wall only approaches the Donnan value without fully establishing a constant potential plateau. In this sense, for the other values of $\bar{\kappa }=6,8,12$ (in dashed lines), the potential decays continuously without reaching the Donnan potential. Finally, for all cases in this figure, the electric potential decays over dimensionless distances of the order of $1/\bar{\kappa }$ on the electrolyte side of the EL/PEL interface, until reaching a practically constant value, where the local electric field is zero [108]. This decay becomes sharper as $\bar{\kappa }$ increases because higher ionic concentrations enhance electrostatic screening and reduce the corresponding EDL thickness, concentrating the potential drop in a narrower region.

**Figure 6:**
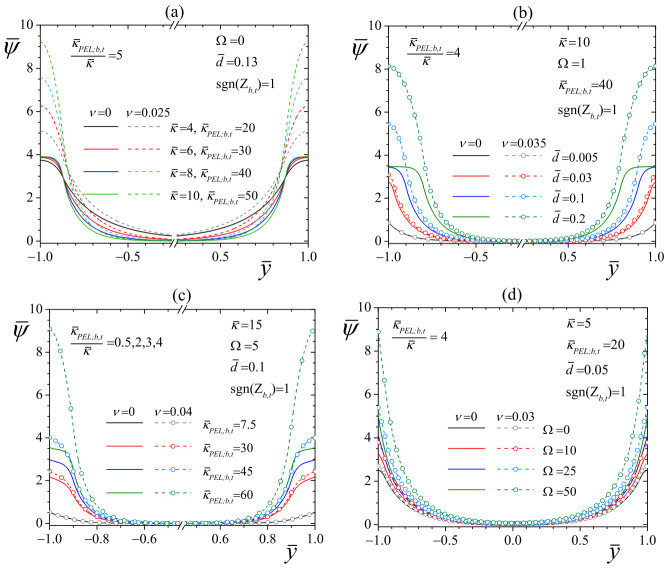
Dimensionless electric potential distribution as a function of the dimensionless transverse coordinate $\bar{y}$, with two steric factor ν values and the symmetric case of $\mathrm{sgn}(Z_{b,t})=1$. (**a**) Effect of increasing the electrokinetic parameters $\bar{\kappa }$ and ${\bar{\kappa }}_{PEL}$ while maintaining a constant ratio of ${\bar{\kappa }}_{PEL}/\bar{\kappa }=5$. (**b**) Effect of PEL thickness $\bar{d}$. (**c**) Effect of electrokinetic parameter ${\bar{\kappa }}_{PEL}$ resulting in increasing values of the ratio ${\bar{\kappa }}_{PEL}/\bar{\kappa }\left(=0.5,2,3,4\right)$. (**d**) Effect of surface charge density Ω.

Figure 6 shows the electric potential distribution $\bar{\psi }$ as a function of the dimensionless transverse coordinate $\bar{y}$, highlighting the influence of the main parameters: the electrokinetic parameters $\bar{\kappa }$ and ${\bar{\kappa }}_{PEL}$, the PEL thickness $\bar{d}$, the increase in the ratio of ${\bar{\kappa }}_{PEL}$ to $\bar{\kappa }$, the steric factor ν, and the surface charge density Ω. In all cases of the figure, $\mathrm{sgn}(Z_{b,t})=1$.

In Figure 6a, the influence of electrokinetic parameters is shown within the ranges $4\leq \bar{\kappa }\leq 10$ and $20\leq {\bar{\kappa }}_{PEL;b,t}\leq 50$, with the combinations shown in the figure for a fixed ratio of ${\bar{\kappa }}_{PEL;b,t}/\bar{\kappa }=5$. The parameters Ω=0 and $\bar{d}=0.13$ remain constant, and the condition $\bar{d}\gg 1/\bar{\kappa }$ is not strictly satisfied. However, for ν=0, the wall potential increases slightly with increasing $\bar{\kappa }$ and ${\bar{\kappa }}_{PEL}$, until it reaches a constant value of ${\bar{\psi }}_{\nu =0}=3.909$. This value approximates the estimated Donnan potential ${\bar{\psi }}_{D}=3.912$, but without establishing a well-defined plateau. In the cases represented by dashed lines with ν=0.025, the wall potential increases with increasing electrokinetic parameters, with ${\bar{\psi }}_{\nu =0.025}=5.08,6.26,7.56,9.23$, corresponding to increments of 23.23%, 20.77%, and 22.09% among them. Here, the potential does not tend to a constant value or plateau, as in the case where the steric effect is absent. In this case, it is not possible to determine the Donnan potential using the mathematical expression discussed in Figure 5 due to the value of ν=0.025. This occurs because the PEL has a limited capacity to hold enough counterions to neutralize its fixed charges and cancel the electrostatic field, even in situations where $\bar{d}\gg 1/\bar{\kappa }$ [108].

Figure 6b analyzes the influence of the PEL thickness $\bar{d}$ on the electric potential distribution while keeping the parameters $\bar{\kappa }=10$, Ω=1, and ${\bar{\kappa }}_{PEL}=40$ constant. When ν=0, increasing the PEL thickness from $\bar{d}=0.005$ to 0.03, 0.1, and 0.2 leads to an increase in surface potentials at $\bar{y}=1$ from ${\bar{\psi }}_{\nu =0}=0.85$ to 2.74, 3.45, and 3.49, respectively. This behavior is due to the fact that a thicker PEL, with more fixed charges, allows for a greater accumulation of free counterions within the PEL, which increases the surface potential [38,74,109]. However, the potential at the walls as a function of $\bar{d}$ stops increasing at an approximate value of ${\bar{\psi }}_{\nu =0}=3.49$, a limit value associated with the Donnan potential. Therefore, in contrast to Figure 5 and Figure 6a, where the Donnan potential arises from the increase in the electrokinetic parameter $\bar{\kappa }$ to fulfill the condition $\bar{d}\gg 1/\bar{\kappa }$, this behavior is now observed through the increase in the PEL thickness $\bar{d}$. Thus, a sufficiently large thickness leads to an excessive accumulation of counterions in the PEL, which shields the fixed charge and cancels the local electric field. Here, the values of $\bar{d}=0.1$ and 0.2 when ν=0 approximate the establishment of the Donnan plateau, whose calculated numerical value is ${\bar{\psi }}_{D}=3.46$. It is evident that the Donnan plateau is not fully established due to the presence of a charged surface imposed by the parameter Ω=1, which generates a nonzero potential gradient near the wall, as observed in the case with $\bar{d}=0.2$. This behavior occurs because the electric field in the vicinity of the surface penetrates partially into the PEL and cannot be fully screened, even when the coating is relatively thick [52]. On the other hand, when ν=0.035, steric exclusion limits the density of counterions available to neutralize the fixed charges, which decreases electrostatic screening and increases the potential near the walls in the PEL region in all cases. For this reason, it is not possible to estimate the Donnan potential because ion packing prevents neutralization of the local electric field. Here, the values of the wall potential in the range of $\bar{d}$ are ${\bar{\psi }}_{\nu =0.035}=0.858,3.088,5.448,8.143$, which correspond to increases of 259.9%, 76.45%, and 49.46% among them, respectively. This behavior highlights the competition between increasing $\bar{d}$, which favors the arising of the Donnan potential, and steric exclusion, which limits ionic accumulation and requires a higher potential difference to mitigate the imbalance between mobile and fixed charges within the PEL. This figure demonstrates that the PEL thickness $\bar{d}$, the surface charge Ω, and the steric factor ν together determine whether the system approaches a Donnan regime or retains a significant potential gradient near the surface.

Figure 6c shows how the electrokinetic parameter of the PEL, denoted as ${\bar{\kappa }}_{PEL;b,t}$, affects the electric potential distribution while keeping $\bar{\kappa }=15$, Ω=5, and $\bar{d}=0.1$ constant. In this case, the condition of $\bar{d}\gg 1/\bar{\kappa }$ is not strictly met. As a result, it lacks an internal region of local electroneutrality necessary to form the Donnan plateau, as in cases with thicker coatings. Under these conditions, and with ν=0, an increase in ${\bar{\kappa }}_{PEL;b,t}$ amplifies the electrostatic effect of the fixed charge within the PEL. This results in a significant increase in the potential at the wall, taking values of ${\bar{\psi }}_{\nu =0}=0.519,2.178,2.981,3.543$ for ${\bar{\kappa }}_{PEL;b,t}=7.5,30,45,60$, respectively. Although the potential at the wall increases with ${\bar{\kappa }}_{PEL;b,t}$, this increase is not proportional since the increases between such values are 319.65%,36.86%, and 18.86% among them, respectively, which shows that screening of the electric field progressively attenuates the effect of the fixed charge as ${\bar{\kappa }}_{PEL;b,t}$ increases. Moreover, it is clear that ratios of ${\bar{\kappa }}_{PEL;b,t}/\bar{\kappa }<1$ result in low surface potentials at the walls, less than unity, while ratios of ${\bar{\kappa }}_{PEL;b,t}/\bar{\kappa }>1$ result in higher electric potential at the walls with values greater than unity due to the increase in the ionic concentration of fixed charges within the PEL. On the other hand, when ν=0.04, steric exclusion limits the local concentration of counterions and reduces the shielding capacity, substantially increasing the wall potential. For example, for ${\bar{\kappa }}_{PEL;b,t}=60$, the wall potential increases from ${\bar{\psi }}_{\nu =0}=3.54$ to ${\bar{\psi }}_{\nu =0.04}=9.08$, which is equivalent to an increase of 156.5% between the two values. Finally, a moderate surface charge density (Ω=5) generates a gradient across the wall that cannot be effectively compensated by a thin PEL.

Figure 6d shows the effect of the surface charge density Ω on the electric potential distribution while keeping $\bar{\kappa }=5$, ${\bar{\kappa }}_{PEL;b,t}=20$, and $\bar{d}=0.05$ constant. Since the PEL thickness satisfies the condition $\bar{d}<1/\bar{\kappa }$, no region of local electroneutrality forms within it, preventing the development of a Donnan potential. In all cases, an increase in Ω results in a higher potential at the wall. For ν=0, the potential increases from ${\bar{\psi }}_{\nu =0}=2.499$ at Ω=0 to ${\bar{\psi }}_{\nu =0}=5.038$ at Ω=50, representing a rise of 101.6% compared to the case with no surface charge. However, for ν=0.03, the steric effects limit the concentration of counterions near the wall, reducing the electrostatic screening effect and leading to significantly higher potentials. In particular, for Ω=50, the wall potential reaches ${\bar{\psi }}_{\nu =0.03}=8.888$, marking a 229.8% increase from the potential of ${\bar{\psi }}_{\nu =0.03}=2.695$ observed at Ω=0. As a result, the case with steric effects demonstrates a more pronounced increase in potential and a greater extension of the charged region towards the channel center. Overall, the figure shows that, for thin PELs ($\bar{d}<1/\bar{\kappa }$), the surface charge density Ω and steric effects ν control the magnitude of the electric potential distribution.

**Figure 7:**
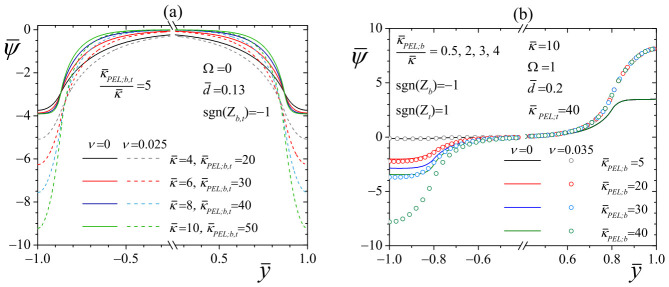
Dimensionless electric potential distribution as a function of the dimensionless transverse coordinate $\bar{y}$ with two steric factor ν values. (**a**) Effect of increasing the electrokinetic parameters $\bar{\kappa }$ and ${\bar{\kappa }}_{PEL}$ while maintaining a constant ratio of ${\bar{\kappa }}_{PEL}/\bar{\kappa }=5$ and with the symmetric case of $\mathrm{sgn}(Z_{b,t})=-1$. (**b**) Effect of electrokinetic parameter ${\bar{\kappa }}_{PEL}$ resulting in increasing values of the ratio ${\bar{\kappa }}_{PEL}/\bar{\kappa }\left(=0.5,2,3,4\right)$ and with the asymmetric case of $\mathrm{sgn}(Z_{b})=-1$ and $\mathrm{sgn}(Z_{t})=1$.

Figure 7a shows how the electrokinetic parameters affect the electric potential distribution when fixed negative charges are present in the PEL, specifically with $\mathrm{sgn}(Z_{b,t})=-1$. This analysis keeps the same ranges for $\bar{\kappa }$ and ${\bar{\kappa }}_{PEL;b,t}$ as in Figure 6a and considers a fixed ratio of ${\bar{\kappa }}_{PEL;b,t}/\bar{\kappa }=5$, with Ω=0 and $\bar{d}=0.13$. As a direct consequence of the reversal of the polarity of the fixed charge, the potential becomes negative, and the profile obtained is a mirror image of that with positive charges. In the absence of the steric effect, with ν=0, the potential inside the PEL reaches a constant value with increasing electrokinetic parameters. With $\bar{\kappa }=10$ and ${\bar{\kappa }}_{PEL;b,t}=50$, the wall potential is ${\bar{\psi }}_{\nu =0}=-3.99$, approaching the formation of a flat region. In particular, when the fixed charged groups correspond to anions, the dimensionless Donnan potential can be estimated by the model proposed by Chanda and Das [41] as ${\bar{\psi }}_{D}=\ln\left\{\left[-\left(1-2\nu \right)+\sqrt{{\left(1-2\nu \right)}^{2}+\left(K_{\lambda }^{4}-4\nu^{2}\right)}\right]/\left(K_{\lambda }^{2}+2\nu \right)\right\}$. Evaluating this expression for ν=0 yields a Donnan potential of ${\bar{\psi }}_{D}=-3.912$, a value that approximates the potential generated with the higher electrokinetic parameters. When the steric effect is present with a value of ν=0.025, the mathematical expression fails to predict the Donnan potential. This is because ionic packing limits ionic accumulation, which reduces the effectiveness of local electric field screening. Thus, the potential magnitude in the PEL region increases in proportions similar to those shown in Figure 6a. This behavior confirms that, as with fixed positive charges, the steric effect reduces the screening of the electric field, requiring a greater potential difference to mitigate the imbalance between mobile and fixed charges within the PEL.

Figure 7b presents the electric potential distribution for an asymmetric system. In this setup, the top wall maintains a fixed ratio of ${\bar{\kappa }}_{PEL;t}/\bar{\kappa }=4$ with a fixed positive charge ($\mathrm{sgn}(Z_{t})=1$). Meanwhile, the bottom wall has a variable ratio of ${\bar{\kappa }}_{PEL;b}/\bar{\kappa }=0.5,\;2,\;3,\;4$ accompanied with fixed negative charges ($\mathrm{sgn}(Z_{b})=-1$). In all cases, $\bar{\kappa }=10$, Ω=1, and $\bar{d}=0.2$ remain constant. This configuration produces asymmetric profiles that evolve toward antisymmetric behavior as the ionic concentration of the fixed negative charge on the bottom wall, denoted as ${\bar{\kappa }}_{PEL;b}$, increases. At the top wall, for ν=0, a Donnan plateau inside the PEL is observed by the solid lines with a value of ${\bar{\psi }}_{D}=3.46$, while the potential at the wall reaches ${\bar{\psi }}_{\nu =0}=3.49$, a difference attributed to the contribution of the surface charge Ω. When the steric effect is included with ν=0.035, the potential at the top wall increases to ${\bar{\psi }}_{\nu =0.035}=8.48$. However, the potential distributions within the PEL do not show a defined internal plateau. The aforementioned is because complete screening of the fixed charges is not possible due to the excluded volume of the ions. On the other hand, at a low ionic concentration of the fixed charge on the bottom wall (${\bar{\kappa }}_{PEL;b}=5$), the profiles for ν=0 and ν=0.035 are practically identical. This is because the electric potential distributions are considered low, within the Debye–Hückel limit, where the steric effect is negligible. For the cases with ν=0, represented by solid lines, the Donnan potential values are predicted by the analytical expression of Chanda and Das [41], yielding ${\bar{\psi }}_{D}=-0.24,-2.09,-2.89,-3.46$ for ${\bar{\kappa }}_{PEL;b}=5,20,30,40$, respectively. In the numerical results presented here, the corresponding electric potential values near the wall and inside the PEL are $\bar{\psi }=-0.14,-2.02,-2.87,-3.46$. It is observed that, for the lowest fixed charge concentration within the PEL, ${\bar{\kappa }}_{PEL;b}=5$, the system does not approach a Donnan regime. In this case, the fixed charge concentration within the PEL is insufficient to induce the complete accumulation of counterions required to establish local electroneutrality, and consequently, an effective electrostatic screening of the electric field inside the layer is not achieved. In contrast, as the fixed charge concentration in the PEL increases for ${\bar{\kappa }}_{PEL;b}=20,30,40$, the enhanced counterion accumulation promotes stronger electrostatic screening and approaches the formation of a local electroneutrality region within the PEL. As a result, the electric potential values closely match the analytically predicted Donnan potentials. It should be noted that the accuracy of the Donnan potential values predicted by Chanda and Das [41] is influenced by the presence of the wall surface charge through the parameter Ω, which introduces an additional electrostatic contribution. For ν=0.035, the predicted Donnan potential values at the bottom wall are ${\bar{\psi }}_{D}=-0.24,-2.35,-3.81$ for ${\bar{\kappa }}_{PEL;b}=5,20,30$, respectively; however, the plateaus are slightly formed by the present solution with approximate values of $\bar{\psi }=-2.24,-3.69$ for ${\bar{\kappa }}_{PEL;b}=20,30$, respectively. For ${\bar{\kappa }}_{PEL;b}=5$, it has a similar value and behavior to the case with ν=0. For ${\bar{\kappa }}_{PEL;b}=40$, the Donnan plateau cannot be estimated using the Chanda and Das mathematical model [41], since the volume excluded from the electrolyte ions produces an insufficient shielding of the local electric field, and therefore the potential in the PEL region tends to increase. Thus, increasing the ratio of ${\bar{\kappa }}_{PEL}/\bar{\kappa }$ leads to higher potentials within the PEL, favoring the formation of the Donnan plateau when ν is absent. These results highlight the critical role of ionic size effects in determining the electrostatic behavior within the PEL, showing that steric constraints can limit charge compensation, prevent the formation of a Donnan equilibrium, and lead to significantly higher electric potentials.

### 4.3. Velocity Profiles

**Figure 8:**
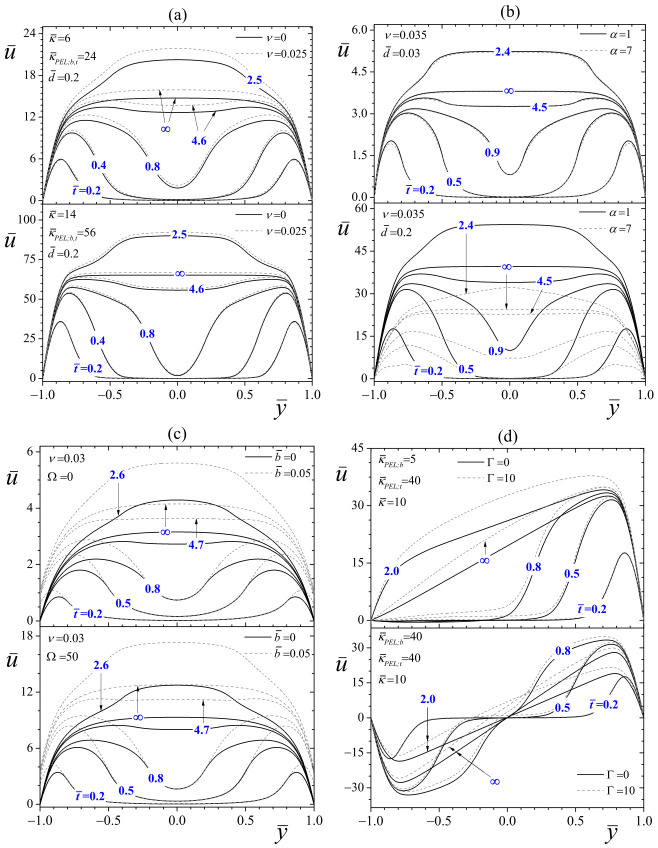
Dimensionless velocity profiles as a function of the dimensionless transverse coordinate $\bar{y}$. (**a**) Effect of the steric factor and the electrokinetic parameters $\bar{\kappa }$ and ${\bar{\kappa }}_{PEL}$, with $\bar{d}=0.2$, Ω=0, Γ=0, α=1, $\bar{b}=0$, $\bar{\lambda }=1$, and $\mathrm{sgn}(Z_{b,t})=1$. (**b**) Effect of drag parameter α and PEL thickness $\bar{d}$, with $\bar{\kappa }=10$, ${\bar{\kappa }}_{PEL;b,t}=40$, ν=0.035, Ω=1, Γ=0, $\bar{b}=0$, $\bar{\lambda }=1$, and $\mathrm{sgn}(Z_{b,t})=1$. (**c**) Effect of the slip length $\bar{b}$ and surface charge density Ω, with $\bar{\kappa }=5$, ${\bar{\kappa }}_{PEL;b,t}=20$, ν=0.03, $\bar{d}=0.05$, α=1, Γ=0, $\bar{\lambda }=1$, and $\mathrm{sgn}(Z_{b,t})=1$. (**d**) Effect of the pressure gradient Γ and ${\bar{\kappa }}_{PEL;b}$, with Ω=1, ${\bar{\kappa }}_{PEL;t}=40$, ν=0.035, $\bar{d}=0.2$, α=1, $\bar{b}$ = 0, $\bar{\lambda }=1$, $\mathrm{sgn}(Z_{b})=-1$, and $\mathrm{sgn}(Z_{t})=1$.

Figure 8 shows the dimensionless velocity profiles, denoted as $\bar{u}$, as a function of the transverse coordinate $\bar{y}$. This figure emphasizes the influence of the electrokinetic parameters $\bar{\kappa }$ and ${\bar{\kappa }}_{PEL}$, the steric factor ν, the PEL thickness $\bar{d}$, the drag parameter α, the surface charge density Ω, the slip length $\bar{b}$, the pressure gradient Γ, and the polarity of the lower wall $\mathrm{sgn}(Z_{b})$ on the flow velocity at different dimensionless times $\bar{t}$. In addition, for this analysis, the relaxation time remains constant at $\bar{\lambda }=1$.

**Figure A1:**
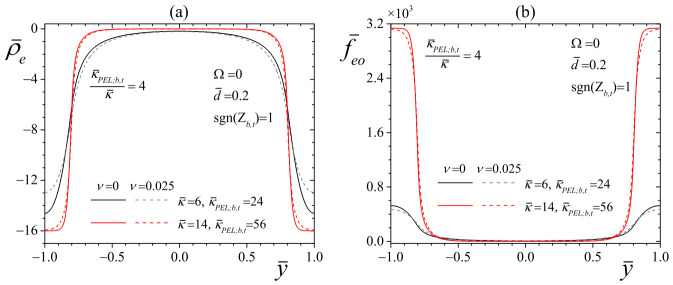
(**a**) Dimensionless free charge density distribution, and (**b**) dimensionless electroosmotic body force, as a function of the dimensionless transverse coordinate $\bar{y}$ with $\mathrm{sgn}(Z_{b,t})=1$, $\bar{d}=0.2$, Ω=0, two values of the electrokinetic parameter $\bar{\kappa }=6,14$ and ${\bar{\kappa }}_{PEL;b,t}=24,56$, and two values of the steric factor ν=0,0.025.

Figure 8a compares the electrokinetic parameters, with $\bar{\kappa }=6$ and ${\bar{\kappa }}_{PEL}=24$ in the upper figure, and $\bar{\kappa }=14$ and ${\bar{\kappa }}_{PEL}=56$ in the lower figure. In both cases, the ratio of ${\bar{\kappa }}_{PEL;b,t}/\bar{\kappa }=4$, and two values of the steric factor ν=0 and ν=0.025 are considered. The velocity profiles shown in this figure originate from the electric potential distributions presented in Figure 5, which correspond to the same combinations of electrokinetic parameters. It is observed that increasing the electrokinetic parameters $\bar{\kappa }$ and ${\bar{\kappa }}_{PEL;b,t}$ results in a substantial increase in the magnitude of the velocity profiles. Taking into account the dimensionless time $\bar{t}=2.5$ as a reference, at which maximum velocities are reached, the velocity at the channel center increases from ${\bar{u}}_{\bar{y}=0}=20.25$ to ${\bar{u}}_{\bar{y}=0}=90.04$; this represents an increase of 344.79% in the absence of the steric effect (ν=0). This significant increase in velocity is not explained solely by a slight increase in the wall potential, but mainly by changes in the distribution of the free charge density and electroosmotic force in the presence of the thick PEL ($\bar{d}=0.2$). Thus, increasing $\bar{\kappa }$ and ${\bar{\kappa }}_{PEL}$ makes the Donnan regime more evident in Figure 5, leading to a less abrupt decay of the electric potential from the channel walls and a more extended potential distribution within the coating. As a result, the free charge density and the electroosmotic body force are distributed over a wider region near the EL/PEL interface, as shown in Figure A1a,b of Appendix A, producing a substantial increase in the velocity profiles. Therefore, the velocity enhancement is primarily governed by the spatial distribution of the free charge density and the electroosmotic force, rather than only by a local increase in wall potential. However, when steric effects are included (ν=0.025), the increase in velocity magnitude is not proportional to that of the electric potential in Figure 5. In this sense, the velocity increases due to the finite ionic size in the steady-state $\bar{t}=\infty$ are relatively small: from ${\bar{u}}_{\bar{y}=0}=14.73$ to ${\bar{u}}_{\bar{y}=0}=15.92$ (8.08%) for the upper figure, and from ${\bar{u}}_{\bar{y}=0}=65.15$ to ${\bar{u}}_{\bar{y}=0}=66.68$ (2.35%) for the lower figure. In this scenario, due to steric effects, the electric potential increases because a larger potential difference is required to counteract the imbalance between the mobile and fixed charges within the PEL (see Figure 5). However, this increase in $\bar{\psi }$ is not proportional to the increase in ${\bar{\rho }}_{e}$, since ionic packing limits the local accumulation of counterions (see Figure A1a). To explain the above, for sufficiently large values of the electric potential, the free charge density reaches a saturation point with ${\bar{\rho }}_{e}\approx -1/\left(2\nu \right)=-20$ for a minimum electric potential given by ${\bar{\psi }}_{\min}\approx \ln\left[(1-2\nu )/\epsilon \nu \right]=8.93$ (with a value of the approximation parameter of ϵ=0.005), as shown in Figure A3 of Appendix A. Although in this case, the steric limit has not been strictly reached (since the maximum values of ${\bar{\rho }}_{e}\approx -15.9$ (see Figure A1a) remain below the saturated value of ${\bar{\rho }}_{e}=-20$); a relatively thick PEL with $\bar{d}=0.2$ and large electrokinetic parameters with $\bar{\kappa }=14$ and ${\bar{\kappa }}_{PEL}=56$ make the distributions of ${\bar{\rho }}_{e}$ and ${\bar{f}}_{eo}$ become similar to those seen without steric effects (see Figure A1a,b). This similarity arises because, under these conditions, the electric potential approaches a Donnan-like regime even in the presence of steric effects (see Figure 5), and the corresponding free charge and electroosmotic force distributions approach the steric saturation limit (see Figure A3 and Figure A4). Thus, the charge distribution is already strongly confined within a narrow region, and the steric effects can no longer produce a noticeable redistribution of ${\bar{\rho }}_{e}$ and ${\bar{f}}_{eo}$. Therefore, the weak increase in velocity observed for ν=0.025 is due to the approximation to a steric saturation condition of the free charge density. In contrast, for the smaller electrokinetic parameters $\bar{\kappa }=6$ and ${\bar{\kappa }}_{PEL}=24$, the distributions of ${\bar{\rho }}_{e}$ and ${\bar{f}}_{eo}$ still show more noticeable differences between ν=0 and ν=0.025, since this condition is further from the steric limit, which explains why the velocity change is slightly larger in the upper figure. Therefore, under thick PEL conditions, the steric effect becomes secondary in the hydrodynamic response because strong electrostatic screening and the approximation to a Donnan plateau limit the charge distribution within the coating.

Figure 8b shows the combined effects of the drag parameter α and the PEL thickness $\bar{d}$ on the dimensionless velocity profiles. In this analysis, the parameters $\bar{\kappa }=10$, ${\bar{\kappa }}_{PEL;b,t}=40$, ν=0.035, Ω=1, and Γ=0 are kept constant. The velocity profiles are generated from the electric potential distributions shown in Figure 6b, particularly for cases with $\bar{d}=0.03$ and $\bar{d}=0.2$ under steric effects ν=0.035. It is observed that increasing the PEL thickness from $\bar{d}=0.03$ to $\bar{d}=0.2$ leads to a substantial increase in the velocity magnitude of 939.6%, from values of ${\bar{u}}_{\bar{y}=0}=5.23$ to ${\bar{u}}_{\bar{y}=0}=54.37$ for $\bar{t}=2.4$ and α=1, which correspond to the maximum velocities in each case. This behavior indicates that a thicker PEL amplifies the electroosmotic flow by increasing both the electric potential near the wall and the effective region where the electroosmotic body force acts, thereby allowing the momentum induced inside the coating to be transmitted over a larger portion of the channel, as discussed in the Appendix A.2. Additionally, when the drag parameter increases from α=1 to α=7, the changes in the magnitude and shape of the velocity profiles become negligible for thin PEL thicknesses, as observed in the upper figure with $\bar{d}=0.03$. In contrast, for a thicker PEL, as shown in the lower figure with $\bar{d}=0.2$, the velocity is attenuated throughout the domain as the parameter α increases. This behavior is attributed to the polyelectrolyte brushes in the coated channel, which increase hydrodynamic friction and dissipate some of the momentum induced by electroosmotic forces. In general, increasing α mainly modulates the flow amplitude. However, a thicker PEL with $\bar{d}=0.2$ also alters the shape of the velocity profile, as the drag force acts over a wider region of the channel. In particular, at flow start-up, the velocity near the wall increases more smoothly for α=7 than for α=1, suggesting less efficient momentum transfer from the PEL to the bulk electrolyte. In steady-state, for the case of $\bar{d}=0.2$, the velocity reduction at the channel center due to the drag effect of the parameter α is from ${\bar{u}}_{\bar{y}=0}=39.57$ to ${\bar{u}}_{\bar{y}=0}=24.44$, representing a decrease of 38.24% for $\bar{t}=\infty$. Finally, the velocity profiles at different dimensionless times show the transient evolution of the flow, in which the central region of nearly uniform velocity gradually consolidates as $\bar{t}$ increases, until reaching the steady-state regime at $\bar{t}=\infty$.

Figure 8c shows the combined effects of the slip length $\bar{b}$ and surface charge density Ω on dimensionless velocity profiles, while keeping $\bar{\kappa }=5$, ${\bar{\kappa }}_{PEL;b,t}=20$, ν=0.03, $\bar{d}=0.05$, α=1, and Γ=0 constant. The velocity profiles in this figure are generated from the electric potential distributions shown in Figure 6d, particularly for the cases with Ω=0 and Ω=50 under steric effects ν=0.03. It is evident that during transient evolution and in the steady-state regime, the presence of hydrodynamic slip ($\bar{b}=0.05$) significantly increases the velocity magnitude throughout the channel domain compared to the case without slip ($\bar{b}=0$). A comparison of the maximum velocities at the channel center for $\bar{t}=2.6$ shows that, in the absence of surface charge (Ω=0), the velocity increases from ${\bar{u}}_{\bar{y}=0}=4.29$ for $\bar{b}=0$ to ${\bar{u}}_{\bar{y}=0}=5.6$ for $\bar{b}=0.05$, which represents an increase of 31.5%. Similarly, in the case with surface charge (Ω=50), the maximum velocity rises from ${\bar{u}}_{\bar{y}=0}=12.74$ to ${\bar{u}}_{\bar{y}=0}=17.31$ when transitioning from a wall without slippage to one with slippage, corresponding to an increase of 35.87%. This behavior is associated with a reduction in wall viscous resistance due to the hydrodynamic slip, enabling more efficient transfer of momentum induced by the electroosmotic force into the fluid. Additionally, increasing the surface charge density significantly enhances flow velocity. For representative values in the steady-state regime and in the presence of slip ($\bar{b}=0.05$), the velocity at the channel center increases from ${\bar{u}}_{\bar{y}=0}=4.15$ for Ω=0 to ${\bar{u}}_{\bar{y}=0}=12.79$ for Ω=50. Physically, a higher value of Ω modifies the electric potential gradient and the spatial distribution of free charge density near the wall, redistributing the electroosmotic forces, which are subsequently transmitted to the fluid through viscous effects. This additional reinforcement results in an overall increase in velocity across the velocity profile. Moreover, the profiles corresponding to different dimensionless times indicate that the flow evolves progressively from a transient state to a steady-state regime. The presence of slip mainly influences the velocity magnitude without significantly changing the transient flow evolution. Notably, with hydrodynamic slip ($\bar{b}=0.05$), the wall velocity is nonzero and exhibits transient behavior during flow start-up. This contrasts with the case without hydrodynamic slip ($\bar{b}=0$), where the no-slip condition is satisfied. The findings indicate that both hydrodynamic slip and surface charge density act as complementary mechanisms that enhance electroosmotic flow by increasing velocity magnitude without significantly altering the overall structure of the velocity profiles.

Figure 8d shows the effect of the pressure gradient Γ on the dimensionless velocity profiles in channels coated with polyelectrolyte layers by keeping Ω=1, $\bar{\kappa }=10$, ν=0.035, $\bar{d}=0.2$, α=1, and $\bar{b}=0$ constant. The velocity profiles originate from the electric potential distributions shown in Figure 7b, where opposite polarities are imposed on the channel walls, with $\mathrm{sgn}(Z_{b})=-1$ at the bottom wall and $\mathrm{sgn}(Z_{t})=1$ at the top wall. In both figures, it is assumed that a positive pressure gradient acts towards the positive flow direction. In the absence of a pressure gradient (Γ=0), the velocity profiles are governed exclusively by electroosmotic effects induced by the surface charge at the walls and within the PELs. In the upper figure, where the fixed charge concentration in the PEL of the bottom wall is relatively low (${\bar{\kappa }}_{PEL;b}=5$), the electroosmotic force is weak. As a result, although the bottom wall has opposite polarity, only small negative velocities are observed, which are limited to the short times of the transient flow stage. In contrast, in the lower figure, where the fixed charge concentration of the PEL at the bottom wall is comparable to that of the top wall ${\bar{\kappa }}_{PEL;b}=40$, the electroosmotic forces acting on both walls have similar magnitudes but act in opposite directions. As a result, antisymmetric velocity profiles for Γ=0 are obtained, with regions of both positive and negative velocities within the channel. Under these conditions, the velocity at the channel center remains zero throughout the transient evolution, leading the velocity profiles at different times to intersect at $\bar{y}=0$. The upper figure shows that a positive pressure gradient (Γ=10) induced by mechanical pumping influences fluid motion during both the transient evolution of velocity and the steady-state regime. As a result, the fluid experiences an increase in velocity throughout the channel domain. On the other hand, in the lower figure, the presence of a pressure gradient with Γ=10 breaks the antisymmetric electroosmotic flow observed for Γ=0, shifting the velocity profile in the positive axial direction and modifying both its magnitude and spatial distribution. In this case, during the transient period, the velocity at the channel center becomes nonzero, and the velocity profiles at different times converge to a position distinct from $\bar{y}=0$. This figure shows the competition between pressure and electroosmotic forces in modulating the magnitude and shape of the velocity profile.

Finally, the set of Figure 8a,b shows flow amplification consistent with previous studies on micro- and nanoscale flows, which have reported significant amplification of fluid transport by incorporating mechanisms such as hydrodynamic slippage and wall coatings [110].

### 4.4. Velocity Tracking

**Figure 9:**
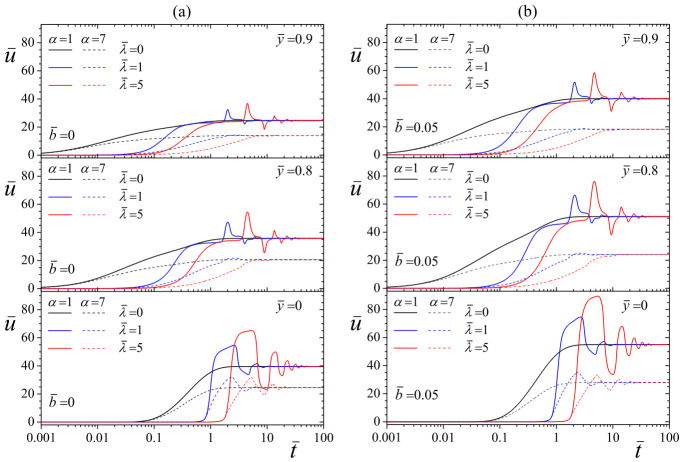
Velocity tracking as a function of the dimensionless time $\bar{t}$ with $\bar{\kappa }=10$, ${\bar{\kappa }}_{PEL}=40$ (${\bar{\kappa }}_{PEL;b,t}/\bar{\kappa }=4$), ν=0.035, $\mathrm{sgn}(Z_{b,t})=1$, $\bar{d}=0.2$, Ω=1, Γ=0, two values of the drag parameter α=1,7, three values of the relaxation time $\bar{\lambda }=0,1,5$, and three positions of the transverse axis $\bar{y}=0,0.8,0.9$. (**a**) $\bar{b}=0$. (**b**) $\bar{b}=0.05$.

Figure 9 shows the velocity tracking as a function of dimensionless time $\bar{t}$ with $\bar{\kappa }=10$, ${\bar{\kappa }}_{PEL;b,t}=40$ producing a ratio of ${\bar{\kappa }}_{PEL;b,t}/\bar{\kappa }=4$, ν=0.035, $\mathrm{sgn}(Z_{b,t})=1$, $\bar{d}=0.2$, Ω=1, Γ=0, two values of the drag parameter α=1,7, three values of the relaxation time $\bar{\lambda }=0,1,5$, and three positions of the transverse axis $\bar{y}=0,0.8,0.9$. For this set of figures, high electric potentials are induced at the walls, as indicated by the green dotted lines with symbols in Figure 6b. The dimensionless time to reach the steady-state regime, denoted as ${\bar{t}}_{ss}$, is determined by analyzing the temporal evolution of velocity at a specified transverse position $\bar{y}$. This is achieved by calculating the velocity deviation, defined as $E_{i}=\left|\bar{u}\left(\bar{y},{\bar{t}}_{i}\right)-\bar{u}\left(\bar{y},{\bar{t}}_{f}\right)\right|$. Here, ${\bar{t}}_{f}\left(=100\right)$ represents the time of the final simulation where the flow is already in steady-state characterized by the absence of temporal variations in velocity, ${\bar{t}}_{i}$ refers to the time prior to ${\bar{t}}_{f}$, and *i* is the time step counter that counts backward from ${\bar{t}}_{f}$ until the condition $E_{i}\geq 10^{-3}$ is satisfied. This threshold marks the transition from the transient state to the steady-state regime, regardless of whether the flow exhibits oscillatory behavior (Maxwell fluids with $\bar{\lambda }\neq 0$) or a monotonic evolution (Newtonian fluids with $\bar{\lambda }=0$). Figure 9a shows the time-tracking of the dimensionless velocity $\bar{u}$ at $\bar{y}=0,0.8$ and 0.9 for a channel without hydrodynamic slippage ($\bar{b}=0$). In the absence of viscoelastic effects ($\bar{\lambda }=0$), the velocity increases monotonically and smoothly to a steady-state regime, exhibiting typical Newtonian fluid behavior governed by the balance of electroosmotic force and viscous resistance. Increasing the relaxation time $\bar{\lambda }$ significantly alters the transient dynamics. For $\bar{\lambda }=1$ and $\bar{\lambda }=5$, overshoots followed by damped oscillations appear, most clearly observed at the channel center ($\bar{y}=0$). This behavior is characteristic of a Maxwell fluid, in which some energy is temporarily stored as elastic stress and released progressively, resulting in oscillatory transient dynamics that gradually converge to the steady-state regime. Quantitatively, the time to reach steady-state increases markedly with $\bar{\lambda }$. For example, for α=1 and $\bar{y}=0$, the times ${\bar{t}}_{ss}$ with $\bar{\lambda }=0$, 1, and 5 are 4.35, 21.27, and 97.36, which show the strong influence of the memory effect on the transient stage of the flow. The steady-state regime is reached more rapidly near the wall (at $\bar{y}=0.8$ and 0.9) than at the center. For $\bar{\lambda }=5$ and α=1, ${\bar{t}}_{ss}$ decreases from 97.36 at $\bar{y}=0$ to 87.58 at $\bar{y}=0.8$ and 85.94 at $\bar{y}=0.9$. Furthermore, at these positions, the transient oscillations exhibit lower amplitude. This is because the region near the wall, where electroosmotic and drag forces within the PEL act directly, favors more efficient dissipation of the stored elastic stress, thereby reducing the time required to reach the steady-state regime. Furthermore, a higher drag (α=7) systematically reduces the time required to reach a steady-state regime. For $\bar{\lambda }=5$, ${\bar{t}}_{ss}$ decreases from 97.36 to 63.63 at $\bar{y}=0$, and from 85.94 to 42.63 at $\bar{y}=0.9$, as shown in Table 2. This behavior indicates that the increased drag acts as an additional viscous resistance, damping the fluid elastic response more rapidly. Taken together, the results show that flow dynamics are strongly modulated by the relaxation time and the resistance mechanisms associated with the drag parameter α in the PEL.

Figure 9b shows the time-tracking of the dimensionless velocity $\bar{u}$ at $\bar{y}=0,0.8$ and 0.9 when hydrodynamic slip is incorporated ($\bar{b}=0.05$). Compared to the case without hydrodynamic slip, the main difference lies in the velocity magnitude. For $\bar{\lambda }=0$, the evolution remains non-oscillatory, but velocity values are consistently higher because of hydrodynamic slippage. With viscoelastic effects ($\bar{\lambda }=1$ and 5), the initial velocity rises more sharply with large overshoots, particularly at the channel center ($\bar{y}=0$). This behavior is associated with the slip condition, which eliminates the strict kinematic constraint $\bar{u}=0$ at the wall and allows the fluid to accelerate more rapidly at first. As a result, the transient response exhibits higher velocities when $\bar{\lambda }>0$. The system then gradually reaches a steady-state regime as the memory effects of the Maxwell model stabilize, balancing the electroosmotic force, the PEL drag force, and the fluid viscous resistance. Quantitatively, hydrodynamic slip significantly amplifies the velocity magnitude, although the times to reach a steady-state remain largely unchanged compared to the case without slip, confirming that the time to reach this regime is dominated by the relaxation time $\bar{\lambda }$ and by the drag α (compare the values in Table 2). For example, for α=1 and $\bar{y}=0$, ${\bar{t}}_{ss}$ evolves from 4.92 ($\bar{\lambda }=0$) to 20.94 ($\bar{\lambda }=1$) and 97.44 ($\bar{\lambda }=5$). Furthermore, increasing the drag (α=7) systematically reduces ${\bar{t}}_{ss}$ at all positions, particularly at $\bar{y}=0.8$ and 0.9, because the polyelectrolyte brushes within the PEL act as an additional damping mechanism. Steady-state is reached earlier in these near-wall regions than at the channel center, although the amplitude of the overshoots remains greater than in the case without slippage. The results indicate that hydrodynamic slippage amplifies flow velocity in both the transient and steady-state regimes, while fluid memory effects and the drag parameter govern the transient stage.

**Figure 10:**
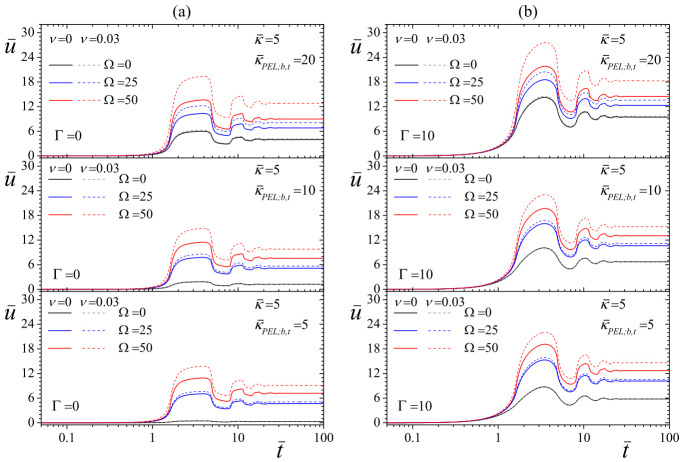
Velocity tracking as a function of the dimensionless time $\bar{t}$ at the center of the channel $\bar{y}=0$, with $\mathrm{sgn}(Z_{b,t})=1$, $\bar{d}=0.05$, $\bar{b}=0.05$, $\bar{\lambda }=2.5$, α=1, two values of the steric factor ν=0,0.03, three values of the surface charge density Ω=0,25,50, and three combinations of the electrokinetic parameter ratios (${\bar{\kappa }}_{PEL;b,t}/\bar{\kappa }=1,2,4$). (**a**) Γ=0. (**b**) Γ=10.

Figure 10 shows the velocity tracking at the channel center ($\bar{y}=0$) as a function of time $\bar{t}$ for different values of the surface charge density Ω, considering the steric effect (ν), three electrokinetic parameter ratios $\left({\bar{\kappa }}_{PEL;b,t}/\bar{\kappa }=1,2,4\right)$, and two pressure gradient conditions Γ=0 and Γ=10.

**Figure A2:**
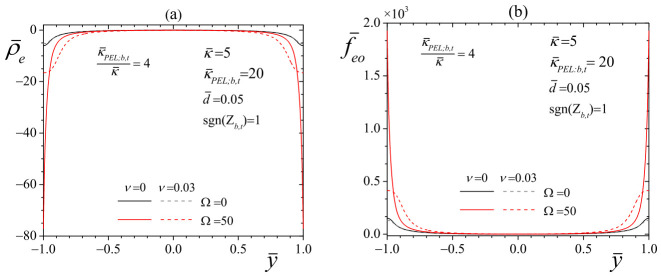
(**a**) Dimensionless free charge density distribution, and (**b**) dimensionless electroosmotic body force, as a function of the dimensionless transverse coordinate $\bar{y}$ with $\mathrm{sgn}(Z_{b,t})=1$, $\bar{d}=0.05$, $\bar{\kappa }=5$ and ${\bar{\kappa }}_{PEL;b,t}=20$, two values of the surface charge density Ω=0,50, and two values of the steric factor ν=0,0.03.

**Figure A3:**
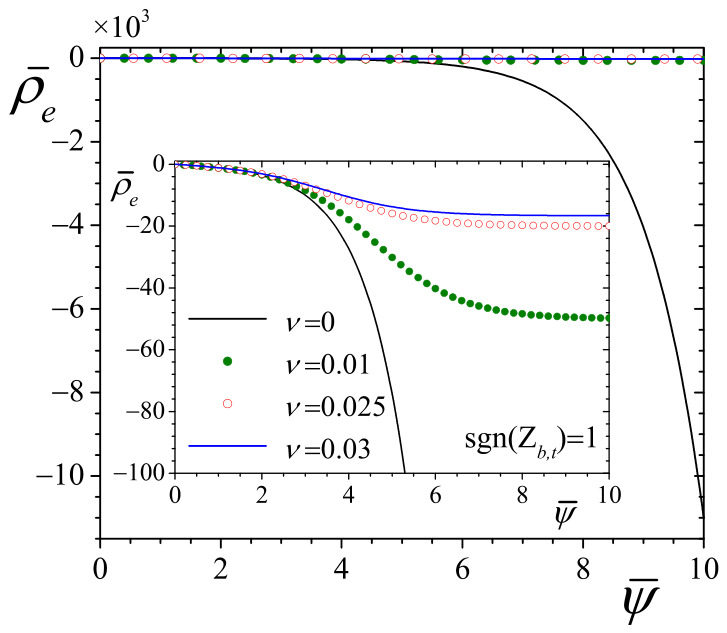
Dimensionless free charge density distribution as a function of the dimensionless electric potential $\bar{\psi }$ with $\mathrm{sgn}(Z_{b,t})=1$, and four values of the steric factor ν=0,0.01,0.025,0.03.

**Figure A4:**
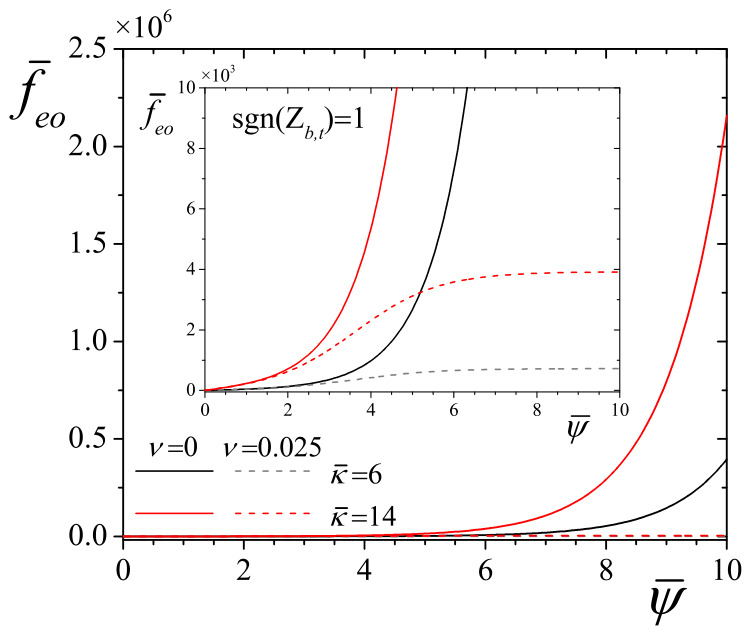
Dimensionless electroosmotic body force as a function of the dimensionless electric potential $\bar{\psi }$ with $\mathrm{sgn}(Z_{b,t})=1$, two values of the electrokinetic parameters $\bar{\kappa }=6,14$, and two values of the steric factor ν=0,0.025.

In Figure 10a, corresponding to the purely electroosmotic case (Γ=0), the increase in surface charge density Ω clearly increases the maximum velocities in the transient and steady-state regimes. This behavior is associated with the increase in the electric potential near the wall (see Figure 6d), which modifies the spatial distribution of the free charge density and enhances the electroosmotic body force. Under the current conditions of a relatively thin PEL ($\bar{d}=0.05$), no Donnan plateau is formed, allowing the electric field to penetrate the region near the walls. Quantitatively, Table 3 shows that Ω also exerts a significant influence on the time required to reach steady-state. In this direction, for ${\bar{\kappa }}_{PEL;b,t}/\bar{\kappa }=1$ and ν=0, the steady-state time increases from ${\bar{t}}_{ss}\approx 28.2$ for Ω=0 to ${\bar{t}}_{ss}\approx 41.64$ for Ω=25 and ${\bar{t}}_{ss}\approx 44.85$ for Ω=50. A similar behavior is maintained for the other electrokinetic ratios, although the sensitivity decreases as Ω and ${\bar{\kappa }}_{PEL;b,t}/\bar{\kappa }$ increase, tending to values between ${\bar{t}}_{ss}\approx 44.85$ and ${\bar{t}}_{ss}\approx 48.13$. On the other hand, the electrokinetic ratio ${\bar{\kappa }}_{PEL;b,t}/\bar{\kappa }$ also significantly modifies the duration of the transient stage, especially when Ω=0. Thus, for ν=0, the time to reach the steady-state increases from ${\bar{t}}_{ss}\approx 28.2$ when ${\bar{\kappa }}_{PEL;b,t}/\bar{\kappa }=1$ to 34.92 when ${\bar{\kappa }}_{PEL;b,t}/\bar{\kappa }=2$ and 41.54 when ${\bar{\kappa }}_{PEL;b,t}/\bar{\kappa }=4$. This indicates that, when the surface charge contribution is weak or zero, the electrokinetic redistribution of the free charge density (counterions) between the PEL region and the electrolyte largely determines the strength of the electroosmotic force and, therefore, the time to reach the steady-state regime. In contrast, for Ω=25 and Ω=50, the differences between the electrokinetic relationships are considerably reduced. Thus, for Ω=25, the times to reach steady-state are ${\bar{t}}_{ss}=41.64$, ${\bar{t}}_{ss}=44.55$, and ${\bar{t}}_{ss}=44.81$, whereas for Ω=50, they are ${\bar{t}}_{ss}=44.85$, ${\bar{t}}_{ss}=44.88$, and ${\bar{t}}_{ss}=47.76$, which correspond to ${\bar{\kappa }}_{PEL;b,t}/\bar{\kappa }=1$, ${\bar{\kappa }}_{PEL;b,t}/\bar{\kappa }=2$, and ${\bar{\kappa }}_{PEL;b,t}/\bar{\kappa }=4$, respectively. The above suggests that, at high surface charges, the electroosmotic force is already strong enough that the effect of ${\bar{\kappa }}_{PEL;b,t}/\bar{\kappa }$ on ${\bar{t}}_{ss}$ becomes secondary. The steric effect has a very limited influence on the time required to reach the steady-state regime. In virtually all cases, the differences between ν=0 and ν=0.03 are relatively small. However, it is observed that the inclusion of the steric effect slightly increases the velocity reached at the channel center, which becomes more noticeable for large values of Ω and the electrokinetic ratio ${\bar{\kappa }}_{PEL;b,t}/\bar{\kappa }$. For example, for Ω=50 and ${\bar{\kappa }}_{PEL;b,t}/\bar{\kappa }=1$, the velocity in steady-state increases from approximately $\bar{u}\approx 7.19$ for ν=0 to $\bar{u}\approx 9.10$ for ν=0.03. For ${\bar{\kappa }}_{PEL;b,t}/\bar{\kappa }=4$, an increase in velocity from approximately $\bar{u}\approx 8.97$ to $\bar{u}\approx 12.78$ is also appreciable. This behavior is consistent with the distributions of free charge density and electroosmotic forces shown in Figure A2a,b of Appendix A, where, for a large value of Ω, steric effects limit the local charge accumulation near the wall while promoting a broader spatial distribution of the electroosmotic forces. As a result, although the local magnitude of the driving force becomes constrained, its effective action extends further into the electrolyte, sustaining the increase in velocity. In particular, for Ω=50, ν=0.03, and ${\bar{\kappa }}_{PEL;b,t}/\bar{\kappa }=4$, the system approaches a regime where the free charge density near the wall is close to its steric saturation limit with ${\bar{\rho }}_{e}\approx -16.66$, as shown in Figure A2a. At this limit of steric saturation, the velocity still increases because the electroosmotic response depends on the spatial redistribution of free charge density and the electroosmotic force generated by the increase in Ω.

In Figure 10b, with Γ=10, the pressure gradient contributes to the dynamics of the electroosmotic flow; thereby, the velocities in the transient and steady-state regimes are higher than in the purely electroosmotic case. Furthermore, overshoot and damped oscillations become more pronounced, indicating a stronger system response to the combined action of electroosmotic forces and external pressure. However, Table 3 shows that the influence of Γ on ${\bar{t}}_{ss}$ is not uniform; that is, its effect is very significant when Ω=0, but becomes much smaller as the surface charge and the ratio ${\bar{\kappa }}_{PEL;b,t}/\bar{\kappa }$ increase. In this sense, for ${\bar{\kappa }}_{PEL;b,t}/\bar{\kappa }=1$ and ν=0, the steady-state time increases from 28.2 at Γ=0 to 41.79 at Γ=10, while for Ω=50, the change is much smaller, from 44.85 to 48.04. For ${\bar{\kappa }}_{PEL;b,t}/\bar{\kappa }=4$, even at Ω=0 the difference is already small, from 41.54 to 45.01. Taken together, these results indicate that the parameters that most affect the time to reach the steady-state are Ω, Γ, and ${\bar{\kappa }}_{PEL;b,t}/\bar{\kappa }$, but their influence is more noticeable at low surface charge densities, particularly when Ω=0.

**Figure 11:**
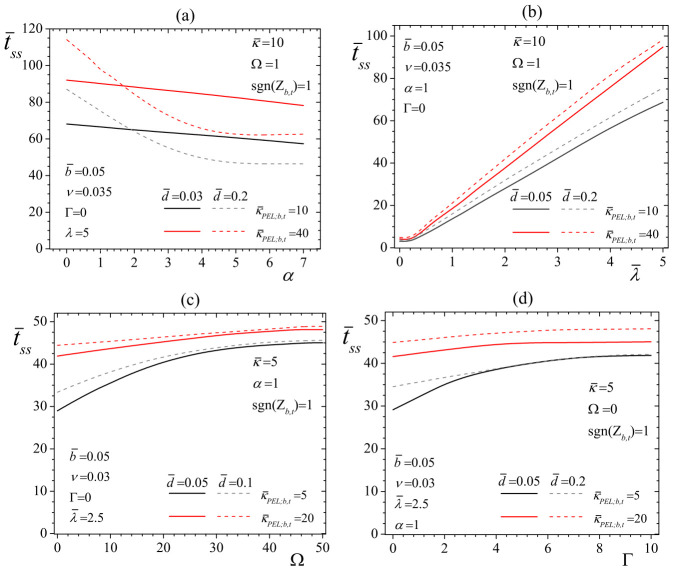
Time to reach the steady-state ${\bar{t}}_{ss}$ of the electroosmotic flow for different dimensionless parameters. (**a**) As a function of the drag parameter α with $\bar{d}\left(=0.03,0.2\right)$ and ${\bar{\kappa }}_{PEL;b,t}\left(=10,40\right)$. (**b**) As a function of the relaxation time $\bar{\lambda }$ with $\bar{d}\left(=0.05,0.2\right)$ and ${\bar{\kappa }}_{PEL;b,t}\left(=10,40\right)$. (**c**) As a function of the surface charge density Ω with $\bar{d}\left(=0.05,0.2\right)$ and ${\bar{\kappa }}_{PEL;b,t}\left(=5,20\right)$. (**d**) As a function of pressure gradient Γ with $\bar{d}\left(=0.05,0.2\right)$ and ${\bar{\kappa }}_{PEL;b,t}\left(=5,20\right)$.

Figure 11 shows how the key parameters influence the dimensionless time needed to reach steady-state, ${\bar{t}}_{ss}$. In Figure 11a, ${\bar{t}}_{ss}$ decreases as the drag parameter α increases, which is attributed to higher hydrodynamic friction. The decrease in ${\bar{t}}_{ss}$ for a thin PEL layer ($\bar{d}=0.03$) is linear, whereas for a thick PEL ($\bar{d}=0.2$), it is more pronounced and significant. This behavior is associated with the transition from a weakly to a heavily damped system as the PEL thickness increases, leading to greater drag forces over a larger region of the channel. For a thick PEL, once the system reaches a highly dissipative regime, further increases in α have a marginal effect on ${\bar{t}}_{ss}$, resulting in a constant slope in the lines. The highest value of the electrokinetic parameter ${\bar{\kappa }}_{PEL;b,t}$ indicates an increase in electroosmotic forces that counteract the drag effect, which increases the transient dynamics time. Figure 11b shows that ${\bar{t}}_{ss}$ increases linearly with the relaxation time $\bar{\lambda }$, indicating that the viscoelastic memory of the Maxwell fluid delays the momentum dissipation during the transient dynamics. As $\bar{\lambda }$ increases, the fluid stores more elastic energy, which is gradually released, thereby extending the damping process of the oscillatory flow. This effect becomes more pronounced for higher values of ${\bar{\kappa }}_{PEL;b,t}$ and $\bar{d}$, where the more intense electroosmotic effects couple with the fluid viscoelasticity, extending the transient regime. Figure 11c shows that ${\bar{t}}_{ss}$ increases with the surface charge density Ω, which occurs because Ω leads to stronger electroosmotic effects that extend the transient dynamics. This effect is further enhanced by increasing ${\bar{\kappa }}_{PEL;b,t}$, which sustains the flow evolution over longer times. However, as Ω increases, its effect on ${\bar{t}}_{ss}$ diminishes because the redistribution of free charge density in the channel is nearly complete. Furthermore, it is observed that the PEL thickness no longer affects ${\bar{t}}_{ss}$ as Ω increases, since both the surface charge density on the wall and the fixed charge density of the PEL play a relevant role in the flow dynamics. Figure 11d shows that ${\bar{t}}_{ss}$ increases with the pressure gradient Γ, exhibiting an effect analogous to Ω in the previous figure. In this context, pressure-driven forces enhance flow dynamics, thereby extending the time required to reach the steady-state. However, as Γ increases and takes on a role similar to that of electroosmotic effects, it ceases to influence ${\bar{t}}_{ss}$. Also, it is observed that, for a combined electroosmotic and pressure-driven flow, the thickness of the PEL layer influences ${\bar{t}}_{ss}$ with high values of ${\bar{\kappa }}_{PEL;b,t}$. Taken together, these graphical results show that time to reach steady-state is governed mainly by the viscoelastic effects $\bar{\lambda }$, friction through the drag parameter α, the PEL thickness, and the ionic concentration of the fixed charge via the electrokinetic parameter of the PEL ${\bar{\kappa }}_{PEL;b,t}$. However, other dimensionless parameters that influence the ${\bar{t}}_{ss}$ but to a lesser extent are the surface charge density Ω and the pressure gradient Γ, whose influence becomes progressively limited as their values tend to be high.

## 5. Conclusions

This study numerically investigated the transient electroosmotic flow of Maxwell fluids in soft parallel-plate channels with polyelectrolyte coatings at high surface potentials, accounting for steric effects, hydrodynamic slip, drag resistance, fixed charge distributions, and pressure gradient contributions. The results showed that electroosmotic transport depends not only on the wall electric potential but also on the spatial distribution of free charge density and electroosmotic forces. Increasing the electrokinetic parameters and the PEL thickness enhances electrostatic screening, promotes the formation of the Donnan plateau, and significantly amplifies the flow by extending the region where electroosmotic forces act. Steric effects limit counterion accumulation and impose a saturation on the free charge density, but they do not suppress the flow; instead, they redistribute the driving forces, particularly in thin PELs. Hydrodynamic slip increases velocity, while drag resistance attenuates it by dissipating momentum within the coating. In addition, the polarity and magnitude of fixed charges can generate asymmetric velocity fields, providing a mechanism for flow control. The interaction between electroosmotic and pressure-driven mechanisms further enriches the dynamics of electroosmotic flow. From a transient perspective, the relaxation time of a Maxwell fluid governs overshoots, damped oscillations, and the time required to reach steady-state, whereas the drag parameter acts as the main dissipative mechanism, accelerating the transition to steady-state. The electrokinetic parameter of the PEL and its thickness delay the time to reach steady-state by strengthening and extending electroosmotic forces. In summary, this work expands the understanding of start-up dynamics and controllability of electroosmotic flow in polyelectrolyte-grafted channels carrying complex rheology fluids under high surface potentials. Furthermore, it provides practical guidelines for the design of advanced micro- and nanofluidic systems, such as lab-on-a-chip and BioMEMS, to address flow enhancement, mixing, drug delivery, diagnostics, fluid management, mass transport, species separation, sample collection, and detection.

## Figures and Tables

**Table 1:** Convergence summary of the mesh independence study based on two representative cases from Figure 3: (a) $\bar{y}=0$, $\bar{t}=4.04$ and (b) $\bar{y}=0$, $\bar{t}=6.96$.

Element Size	Number of Elements	(a) $\bar{u}\left(\bar{y},\bar{t}\right)$	$\varepsilon_{a,i}\;\left(\%\right)$	(b) $\bar{u}\left(\bar{y},\bar{t}\right)$	$\varepsilon_{a,i}\;\left(\%\right)$
0.4	15	69.44928		33.83851	
0.1	27	67.70217	2.58058	34.60194	2.20633
0.009	222	67.92608	0.32964	34.47525	0.36747
**0.008**	**251**	**67.92649**	**0.0006**	**34.47505**	**0.00058**
0.002	1000	67.92658	0.00013	34.47476	0.00085

**Table 2:** Dimensionless time ${\bar{t}}_{ss}$ to reach the steady-state regime, corresponding to Figure 9, evaluated at different relaxation times $\bar{\lambda }$, drag coefficients α, and transverse locations $\bar{y}$, for two slip parameters $\bar{b}=0$ and $\bar{b}=0.05$.

$\bar{\lambda }$	$\bar{y}=0$		$\bar{y}=0.8$		$\bar{y}=0.9$	
	α=1	α=7	α=1	α=7	α=1	α=7
	$\bar{b}=0$ / $\bar{b}=0.05$	$\bar{b}=0$ / $\bar{b}=0.05$	$\bar{b}=0$ / $\bar{b}=0.05$	$\bar{b}=0$ / $\bar{b}=0.05$	$\bar{b}=0$ / $\bar{b}=0.05$	$\bar{b}=0$ / $\bar{b}=0.05$
0	4.35/4.92	3.59/3.71	3.88/4.47	3.06/3.18	3.6/4.25	2.74/2.9
1	21.27/20.94	16.01/15.87	19.5/18.71	12.82/12.8	17.67/18.6	12.52/12.49
5	97.36/97.44	63.63/63.68	87.58/87.38	49.26/49.25	85.94/86.77	42.63/42.76

**Table 3:** Dimensionless time ${\bar{t}}_{ss}$ to reach the steady-state regime, corresponding to Figure 10, evaluated at different surface charge densities Ω, steric factor ν, three combinations of the electrokinetic parameter ratios, and for two pressure gradient values Γ=0 and Γ=10.

Ω	${\bar{\kappa }}_{\mathrm{PEL};b,t}/\bar{\kappa }=1$		${\bar{\kappa }}_{\mathrm{PEL};b,t}/\bar{\kappa }=2$		${\bar{\kappa }}_{\mathrm{PEL};b,t}/\bar{\kappa }=4$	
	ν=0	ν=0.03	ν=0	ν=0.03	ν=0	ν=0.03
	Γ=0 / Γ=10	Γ=0 / Γ=10	Γ=0 / Γ=10	Γ=0 / Γ=10	Γ=0 / Γ=10	Γ=0 / Γ=10
0	28.2/41.79	28.2/41.79	34.92/44.54	34.93/44.55	41.54/45.01	41.57/45.03
25	41.64/45.07	44.51/45.1	44.55/45.1	44.65/47.78	44.81/48	44.92/48.11
50	44.85/48.04	45/48.18	44.88/48.07	47.86/48.22	47.76/48.18	48.13/48.37

## Data Availability

The original contributions presented in this study are included in the article. Further inquiries can be directed to the corresponding author.

## Nomenclature

*a*	Effective ionic size	m
*b*	Slip length	m
$\bar{b}$	Dimensionless slip length	-
*d*	PEL thickness	m
$\bar{d}$	Dimensionless PEL thickness	-
*e*	Elementary charge	C
$E_{x}$	Axial electric field	V $m^{-1}$
$E_{i}$	Velocity deviation	-
E	Electric field vector	V $m^{-1}$
$E_{i}$	Velocity deviation	-
$f_{eo}$	Electroosmotic body force	N $m^{-3}$
$f_{eo,c}$	Characteristic electroosmotic body force	N $m^{-3}$
${\bar{f}}_{eo}$	Dimensionless electroosmotic body force	-
$k_{B}$	Boltzmann constant	J $K^{-1}$
*k*	Friction coefficient	Pa s $m^{-2}$
*L*	Length of the channel	m
*M*	Molar concentration of the background electrolyte solution	mol $L^{-1}$
*m*	Total number of data points	-
$n_{0}$	Ionic number concentration in the bulk solution	$m^{-3}$
$n_{+}$	Number concentration of positive ions	$m^{-3}$
$n_{-}$	Number concentration of negative ions	$m^{-3}$
*N*	Ionic number concentration of fixed charges within the PEL	$m^{-3}$
*p*	Pressure	Pa
$p_{x}$	External pressure gradient	Pa $m^{-1}$
*q*	Nodal counter	-
*t*	Time	s
$\bar{t}$	Dimensionless time	-
${\bar{t}}_{f}$	Dimensionless time of the final simulation	-
${\bar{t}}_{i}$	Dimensionless time *i* before the final simulation	-
${\bar{t}}_{ss}$	Dimensionless time required to reach steady-state	-
*T*	Temperature	K
*u*	Axial velocity	m $s^{-1}$
$u_{c}$	Characteristic velocity	m $s^{-1}$
${\bar{u}}_{i}$	Current dimensionless velocity for mesh *i*	-
${\bar{u}}_{j}$	Previous dimensionless velocity for mesh *j*	-
$\bar{u}$	Dimensionless axial velocity	-
v	Velocity vector	m $s^{-1}$
x,y	Cartesian coordinates	m
$\bar{y}$	Dimensionless transverse coordinate	-
*z*	Valence of electrolyte ions	-
*Z*	Valence of the fixed ions within the PEL	-
*Greek symbols*		
α	Drag parameter	-
Γ	Dimensionless pressure gradient	-
$\dot{\gamma }$	Rate-of-strain tensor	$s^{-1}$
ε	Dielectric permittivity of medium	C $V^{-1}m^{-1}$
$\varepsilon_{a,i}$	Approximate relative error	%
$\varepsilon_{s}$	Convergence rate	%
$\eta_{0}$	Zero-shear-rate viscosity	Pa s
$\kappa^{-1}$	Debye length in the electrolyte layer	m
$\bar{\kappa }$	Electrokinetic parameter of the electrolyte layer	-
$\kappa_{PEL}^{-1}$	Equivalent Debye length in the PEL	m
${\bar{\kappa }}_{PEL}$	Electrokinetic parameter of the PEL	-
λ	Relaxation time	s
$\bar{\lambda }$	Dimensionless relaxation time	-
ν	Steric factor	-
ρ	Fluid density	kg $m^{-3}$
$\rho_{e}$	Volumetric free charge density	C $m^{-3}$
${\bar{\rho }}_{e}$	Dimensionless volumetric free charge density	-
$\rho_{PEL}$	Volumetric fixed charge density in the PEL	C $m^{-3}$
σ	Surface charge density	C $m^{-2}$
$\tau_{yx}$	Shear stress	Pa
${\bar{\tau }}_{yx}$	Dimensionless shear stress	-
τ	Stress tensor	Pa
ψ	Electric potential in the EDL	V
$\bar{\psi }$	Dimensionless electric potential in the EDL	-
Ω	Dimensionless surface charge density	-

## Appendix A. Free Charge Density and Electroosmotic Body Force

In the electroosmotic flow considered in this work, the driving mechanism originates from the interaction between the applied electric field and the free charge density distributed near the channel walls, generating the electroosmotic body force $\rho_{e}E$ that drives the fluid, as described in the momentum Equations (3) and (5). Since the electric potential distribution determines the free charge density, it is useful to derive its dimensionless form and the corresponding body force.

### Appendix A.1. Mathematical Derivation of the Free Charge Density and Electroosmotic Body Force

The free charge density $\rho_{e}$ is defined as:

(A1) $$\rho_{e}=ez\left(n_{+}-n_{-}\right),$$

where $n_{+}$ and $n_{-}$ are the number concentrations of positive and negative ions, respectively. When steric effects are included, the ion distributions follow the modified Boltzmann distribution of Equation (7). Substituting Equation (7) to the definition of $\rho_{e}$ in Equation (A1) yields:

(A2) $$\rho_{e}=-2zen_{0}\frac{\sinh(ze\psi /k_{B}T)}{1+2\nu \left[\cosh(ze\psi /k_{B}T)-1\right]}.$$

On the other hand, the electroosmotic body force is expressed as $f_{eo}=\rho_{e}E_{x}$. By using Equation (A2), and the definition of $\kappa^{2}=2z^{2}e^{2}n_{0}/\varepsilon k_{B}T$, which results in $2zen_{0}=\varepsilon k_{B}T\kappa^{2}/ze$, the electroosmotic body force becomes:

(A3) $$f_{eo}=-\frac{\varepsilon k_{B}T\kappa^{2}}{ze}\frac{\sinh(ze\psi /k_{B}T)}{1+2\nu \left[\cosh(ze\psi /k_{B}T)-1\right]}E_{x}.$$

To obtain the dimensionless form of the free charge density from Equation (A2) and the electroosmotic body force from Equation (A3), they are normalized using the following dimensionless variables:

(A4) $${\bar{\rho }}_{e}=\frac{\rho_{e}}{2zen_{0}},\;{\bar{f}}_{eo}=\frac{f_{eo}}{f_{eo,c}},$$

where the characteristic electroosmotic body force is obtained using the definition of the characteristic velocity described in Section 2.3, resulting in $f_{eo,c}=\eta_{0}u_{c}/H^{2}=-\varepsilon k_{B}TE_{x}/zeH^{2}$. Introducing the dimensionless variables from Equation (A4) and the dimensionless definition of the electric potential $\bar{\psi }=ze\psi /k_{B}T$ from Equation (17), the dimensionless free charge density becomes:

(A5) $${\bar{\rho }}_{e}=-\frac{\sinh(\bar{\psi })}{1+2\nu \left[\cosh(\bar{\psi })-1\right]},$$

and the dimensionless electroosmotic body force is:

(A6) $${\bar{f}}_{eo}={\bar{\kappa }}^{2}\frac{\sinh(\bar{\psi })}{1+2\nu \left[\cosh(\bar{\psi })-1\right]}.$$

In the absence of steric effects (ν=0), the denominator in Equations (A5) and (A6) reduces to unity. Therefore, the free charge density is expressed as:

(A7) $${\bar{\rho }}_{e}=-\sinh\left(\bar{\psi }\right),$$

and the electroosmotic body force is:

(A8) $${\bar{f}}_{eo}={\bar{\kappa }}^{2}\sinh\left(\bar{\psi }\right),$$

which implies that both the free charge density and the electroosmotic body force increase exponentially with the electric potential. However, when ν≠0, the denominator grows with $\cosh(\bar{\psi })$ and competes with the numerator. For sufficiently large electric potentials $\bar{\psi }\gg 1$, the hyperbolic functions can be approximated as:

(A9) $$\sinh\left(\bar{\psi }\right)\approx \frac{e^{\bar{\psi }}}{2},\;\cosh\left(\bar{\psi }\right)\approx \frac{e^{\bar{\psi }}}{2}.$$

Substituting these expressions into Equation (A5) of the free charge density yields:

(A10) $${\bar{\rho }}_{e}\approx -\frac{\frac{1}{2}e^{\bar{\psi }}}{1+\nu e^{\bar{\psi }}-2\nu }.$$

When the exponential term dominates the denominator, i.e., when

(A11) $$\nu e^{\bar{\psi }}\gg \left(1-2\nu \right),$$

the constant term becomes negligible and the charge density approaches a limiting charge saturation value given by:

(A12) $${\bar{\rho }}_{e}\approx -\frac{1}{2\nu }.$$

This result indicates that the free charge density saturates as the electric potential increases. Therefore, although the electric potential within the polyelectrolyte layer may increase significantly, the electroosmotic body force is not proportional to this increase, as the free charge density approaches a constant value determined by the steric factor. A quantitative criterion for the validity of this approximation can be obtained by imposing:

(A13) $$\nu e^{\bar{\psi }}\geq m\left(1-2\nu \right),$$

where the parameter *m* indicates how dominant the term $\nu e^{\bar{\psi }}$ is with respect to the constant term. Solving for the potential yields:

(A14) $$\bar{\psi }\geq \ln\left(\frac{m(1-2\nu )}{\nu }\right).$$

Alternatively, the free charge density can be expressed in terms of a prescribed small parameter ϵ that controls the validity of the approximation at large electric potentials. In this direction, Equation (A10) can be rewritten as:

(A15) $${\bar{\rho }}_{e}=-\frac{1}{2\nu }\left(\frac{1}{1+\frac{1-2\nu }{\nu e^{\bar{\psi }}}}\right),$$

where it can be observed that the term that controls the error is:

(A16) $$\delta =\frac{1-2\nu }{\nu e^{\bar{\psi }}},$$

and imposing δ≤ϵ:

(A17) $${\bar{\psi }}_{\min}\approx \ln\left(\frac{1-2\nu }{\epsilon \nu }\right),$$

which defines the minimum electric potential required to reach the saturation regime of the free charge density for given values of ν and ϵ. Under this condition, the free charge density approaches its limiting value ${\bar{\rho }}_{e}\approx -1/\left(2\nu \right)$, indicating that further increases in the electric potential do not produce a proportional increase in ${\bar{\rho }}_{e}$ due to steric effects.

### Appendix A.2. Physical Interpretation of Charge Saturation

To clarify the physical mechanisms that govern the velocity variations observed in Figure 8a, Figure A1 presents the distributions of the dimensionless free charge density and the electroosmotic body force obtained from the electric potentials shown in Figure 5, for the two extreme cases $\left(\bar{\kappa },{\bar{\kappa }}_{PEL;b,t}\right)=\left(6,24\right)$ and (14,56), since these potentials generate the velocity profiles shown in Figure 8a.

In this regard, Figure A1a shows the dimensionless distribution of free charge density, ${\bar{\rho }}_{e}\left(\bar{y}\right)$, along the channel. Near the wall, inside the PEL, the steric effect reduces the magnitude of the free charge density. For example, for $\bar{\kappa }=6$ and ${\bar{\kappa }}_{PEL;b,t}=24$, ${\bar{\rho }}_{e}$ decreases from ${\bar{\rho }}_{e}=-14.61$ for ν=0 to ${\bar{\rho }}_{e}=-12.97$ for ν=0.025. This behavior is due to the inclusion of steric effects in the modified Boltzmann distribution, where the denominator term $1+2\nu [\cosh\left(\bar{\psi }\right)-1]$ competes with the numerator term $\sinh(\bar{\psi })$ and limits the space available for counterion accumulation, thus preventing an unlimited increase in charge density. In addition to this local reduction, the steric effect redistributes the free charge density over a wider region near the EL/PEL interface, modifying the spatial extent of the electroosmotic forces. Conversely, as ν decreases, the system approaches the classical pointlike charges, where the steric restriction weakens, allowing more counterions to accumulate in the PEL region and increasing the free charge density. However, for the larger electrokinetic parameters, $\bar{\kappa }=14$ and ${\bar{\kappa }}_{PEL;b,t}=56$, the free charge density is similar with or without the steric effect, with ${\bar{\rho }}_{e}=-15.99$ for ν=0 and ${\bar{\rho }}_{e}=-15.92$ for ν=0.025. This behavior can be attributed to two reasons. First, in the absence of steric effects, the electric potential within the PEL approaches a Donnan plateau, thereby limiting further increases in the free charge density, as discussed in Section 4.2. And second, when steric effects are included, the charge density approaches a saturation level of the order of ${\bar{\rho }}_{e}\approx -1/\left(2\nu \right)\approx -1/\left(2\times 0.025\right)=-20$. Therefore, in both cases, the free charge density is restricted, albeit through different physical mechanisms. Additionally, outside the PEL region, the behavior differs slightly. In this region, the steric effect produces a small increase in the free charge density compared with the case ν=0. This occurs because ion redistribution due to steric restrictions alters electrostatic shielding, allowing a slightly greater amount of free charge to remain in the electrolyte region. Consequently, the steric effect not only reduces the charge density inside the PEL region but also redistributes it in the electrolyte region. Figure A1b shows the corresponding distribution of the dimensionless electroosmotic body force along the channel. Since the electroosmotic force is proportional to the product of the free charge density and the electric field, its spatial distribution follows the regions of charge accumulation. In the PEL region, the force reaches its largest magnitude due to the strong coupling between the electric potential gradient and the free charge density. As the electrokinetic parameters increase from $\left(\bar{\kappa },{\bar{\kappa }}_{PEL;b,t}\right)=\left(6,24\right)$ to (14,56), the electroosmotic force becomes significantly stronger within the coating, which directly contributes to the large increase in the velocity profiles observed in Figure 8a. However, when steric effects are present (ν=0.025), the increase in the electroosmotic force is less pronounced than the corresponding increase in the electric potential. This occurs because the free charge density becomes limited by the steric effect, as discussed in Figure A1a. Consequently, although the electric potential inside the PEL increases under steric effects, the electroosmotic force does not increase proportionally. This behavior explains why the steric effect plays a secondary role in the velocity response when large electrokinetic parameters generate high electrical potentials.

Figure A2 illustrates the influence of the surface charge density Ω and the steric factor ν on the distributions of the free charge density and the electroosmotic body force. The results are obtained from the electric potential distributions shown in Figure 6d for a relatively thin PEL thickness $\bar{d}=0.05$, considering two representative values of the surface charge density, Ω=0 and Ω=50, and two steric conditions, ν=0 and ν=0.03.

Figure A2a shows the distribution of the dimensionless free charge density ${\bar{\rho }}_{e}\left(\bar{y}\right)$. For Ω=0, the electric potential remains relatively low, and the corresponding charge density distributions for ν=0 and ν=0.03 are nearly identical, indicating that steric effects are negligible for these values. In contrast, for Ω=50, the electric potential increases significantly (see Figure 6d), leading to a strong accumulation of free charges near the wall when ν=0. When the steric effect is included with ν=0.03, the free charge density reaches a saturation level of approximately ${\bar{\rho }}_{e}∼-1/\left(2\nu \right)\approx -16.66$. This saturation occurs at an estimated minimum potential of ${\bar{\psi }}_{\min}\approx 8.74$, with ϵ=0.005, which is close to the maximum value of the estimated potential at the wall, as is shown in Figure 6d. As a result, the local increase of ${\bar{\rho }}_{e}$ is limited, causing the charge distribution to extend more into the channel interior. This behavior is observed under thin PEL conditions ($\bar{d}=0.05$), where a Donnan plateau is not developed and the local electric field is not screened. This lack of screening allows the redistribution of the electroosmotic force, significantly influencing the overall flow response.

Figure A2b presents the corresponding electroosmotic body force distributions. While steric effects reduce the peak value of ${\bar{f}}_{eo}$ near the wall, they also increase its spatial extent, thereby broadening the region over which electroosmotic forces act. This redistribution explains why, under thin PEL conditions, flow enhancement occurs despite the local saturation of the free charge density. Therefore, the electroosmotic response depends on the overall spatial distribution of ${\bar{f}}_{eo}$ rather than only on its maximum value. As a result, the electroosmotic force transitions from a highly localized behavior near the wall to a more spatially distributed one.

Figure A3 shows the dimensionless free charge density ${\bar{\rho }}_{e}$ as a function of the electric potential $\bar{\psi }$ for different values of the steric factor ν. In the classical limit (ν=0), the charge density follows the Boltzmann relation given in Equation (A7), which leads to an exponential increase in magnitude as the electric potential grows. When steric effects are included (ν>0), the increase in charge density becomes progressively limited due to the finite size of the ions [55]. In this case, the modified Boltzmann distribution predicts a saturation of the free charge density on the order of ${\bar{\rho }}_{e}∼-1/\left(2\nu \right)$. This behavior can be quantified using Equation (A17), which provides an estimate of the electric potential required for steric saturation. For example, for ν=0.03 and ϵ=0.005, the predicted threshold potential is ${\bar{\psi }}_{min}\approx 8.74$. For electric potentials larger than this value, the free charge density approaches the limiting value ${\bar{\rho }}_{e}\approx -1/\left(2\nu \right)$ and remains nearly constant, as observed in this Figure A3. This result confirms that the steric effect imposes a maximum packing limit on counterions, preventing the unlimited growth of the charge density predicted by the classical model.

Figure A4 illustrates the dependence of the dimensionless electroosmotic body force on the electric potential according to Equation (A6). Since the electroosmotic force is proportional to the free charge density, its behavior follows the trends observed for ${\bar{\rho }}_{e}$ in Figure A3. In the classical case (ν=0), the electroosmotic force increases rapidly with the electric potential because the free charge density grows according to the Boltzmann relation. When steric effects are included (ν≠0), the increase of ${\bar{f}}_{eo}$ with $\bar{\psi }$ becomes progressively limited due to the steric correction appearing in the denominator of Equation (A6). This limitation arises from the saturation of the free charge density, which approaches the value ${\bar{\rho }}_{e}\approx -1/\left(2\nu \right)$ for large electric potentials. Consequently, although increasing $\bar{\psi }$ enhances the electroosmotic driving force, the steric constraint prevents an unlimited amplification of ${\bar{f}}_{eo}$. Additionally, the electrokinetic parameter $\bar{\kappa }$ scales the magnitude of the electroosmotic body force through the factor ${\bar{\kappa }}^{2}$, which explains the larger values observed for $\bar{\kappa }=14$ compared to $\bar{\kappa }=6$.
